# Constitutive Expression of AtVTE2 in Flax Is Associated with Changes in Isoprenoid Metabolism and Stem Cell-Wall Composition

**DOI:** 10.3390/ijms27188295

**Published:** 2026-09-17

**Authors:** Karolina Hasiewicz-Derkacz, Iwan Zalewski, Aleksandra Boba, Lucyna Dymińska, Dzmitry Lysh, Tadeusz Czuj, Anna Prescha, Katarzyna Skórkowska-Telichowska, Jan Szopa, Anna Kulma

**Affiliations:** 1 Department of Genetic Biochemistry, Faculty of Biotechnology, University of Wroclaw, Przybyszewskiego 63, 51-148 Wrocław, Polandaleksandra.boba@uwr.edu.pl (A.B.); szopa@ibmb.uni.wroc.pl (J.S.); 2 Department of Dietetics and Bromatology, Wroclaw Medical University, Borowska 211, 50-556 Wrocław, Poland; iwan.zalewski@umw.edu.pl (I.Z.); anna.prescha@umw.edu.pl (A.P.); 3 Department of Bioorganic Chemistry, Faculty of Production Engineering, Wroclaw University of Economics and Business, Komandorska 118/120, 53-345 Wrocław, Poland; lucyna.dyminska@ue.wroc.pl; 4 Medical Faculty, Wrocław University of Science and Technology, Grunwaldzki 11, 51-377 Wrocław, Poland; katarzyna.skorkowska-telichowska@upwr.edu.pl; 5 University College of Professional Education in Wroclaw, Plac Powstańców Śląskich 1, 53-329 Wrocław, Poland

**Keywords:** *Linum usitatissimum*, vitamin E biosynthesis, tocopherol, metabolic engineering, isoprenoid metabolism, phenylpropanoid metabolism, phytosterols, cell wall, lignification, antioxidant capacity

## Abstract

Tocopherols are essential lipid-soluble antioxidants that play important roles in plant protection against oxidative stress and are closely connected with isoprenoid metabolism. To the best of our knowledge, this is the first report of stable constitutive expression of *Arabidopsis thaliana VTE2* (*AtVTE2*) in flax (*Linum usitatissimum* L.) and its effects on plant metabolism and development. As a dual-purpose oil and fibre crop, flax combines PUFA-rich seeds with industrially important fibre-rich stems, providing a useful system for assessing the broader consequences of antioxidant-pathway engineering. Three independent transgenic lines were characterized by RT-qPCR analysis of genes associated with tocopherol, MEP, MVA, and carotenoid biosynthesis, targeted metabolite profiling, antioxidant-capacity assays, field phenotyping, and biochemical and spectroscopic analyses of mature straw. Three independent transgenic lines were characterized using gene-expression analyses, metabolic profiling, antioxidant assays, phenotypic evaluation, and biochemical analyses of mature stems. Constitutive *AtVTE2* expression was associated with α-tocopherol accumulation and was accompanied by coordinated transcriptional changes in genes associated with the plastidial methylerythritol phosphate and cytosolic mevalonate pathways. These changes were associated with modifications in isoprenoid and phenylpropanoid metabolism, enhanced antioxidant capacity in selected transgenic lines, and altered plant architecture under field conditions. In mature straw, transgenic plants exhibited changes in phytosterol composition, lignin accumulation, and pectin distribution, whereas cellulose content remained unchanged. FT-IR spectra retained the same principal bands but showed differences in the relative intensities of selected cell-wall-associated bands. Collectively, these findings demonstrate that constitutive *AtVTE2* expression is associated with tissue-dependent metabolic responses extending beyond vitamin E accumulation and affecting interconnected pathways involved in secondary metabolism, antioxidant capacity, and cell-wall organization.

## 1. Introduction

Tocopherols, collectively known as vitamin E, are lipophilic antioxidants synthesized by photosynthetic organisms and localized predominantly in plastid membranes, including thylakoids, chloroplast envelopes and plastoglobuli. In green tissues, they protect polyunsaturated membrane lipids by quenching singlet oxygen, scavenging lipid-derived radicals and interrupting lipid-peroxidation chains. Tocopherols also protect storage lipids and contribute to seed longevity during dormancy and germination [1,2]. Tocopherol biosynthesis occurs in plastids through the condensation of homogentisate with phytyl diphosphate. This committed reaction is catalyzed by homogentisate phytyltransferase, also known as HPT or VTE2, and produces 2-methyl-6-phytyl-1,4-benzoquinol (MPBQ). Subsequent methylation and cyclization reactions catalyzed by VTE3 and VTE1 generate δ- and γ-tocopherol intermediates, which are further methylated by VTE4 to form β- and α-tocopherol, respectively, thereby shaping the final tocopherol homologue profile. HPT frequently exerts substantial control over total pathway flux, but precursor availability and downstream methylation can restrict the accumulation of individual tocopherol homologues [3,4]. VTE2 has therefore been widely used as a target for vitamin E biofortification. Its overexpression increased tocopherol accumulation in *Arabidopsis*, lettuce and tomato, although the magnitude and composition of the response differed among species and organs [3,5]. These variable outcomes indicate that increased VTE2 activity does not alone determine the final metabolic phenotype and that the effects of the modification depend on the regulation of the surrounding metabolic network.

Tocopherol synthesis relates to broader plastidial metabolism through the supply of phytyl diphosphate and homogentisate. Phytyl diphosphate can originate from plastidial isoprenoid biosynthesis or from phytol released during chlorophyll turnover, linking tocopherol formation with chlorophyll and carotenoid homeostasis. In addition, although the plastidial MEP and cytosolic MVA pathways are spatially separated, compensatory regulation and conditional exchange of intermediates between them allow perturbations in one compartment to influence isoprenoid metabolism in the other [6,7]. Consequently, engineering tocopherol synthesis may have metabolic effects extending beyond the immediate pathway.

Changes in tocopherol metabolism may also interact with other antioxidant and structural components of plant tissues. Manipulation of tocopherol-pathway enzymes in *Arabidopsis* altered ascorbate and glutathione pools, whereas differences in tocopherol composition under salinity were associated with changes in phenolic-acid profiles, pectin epitopes and cell-wall mechanical properties [8,9]. These observations suggest that the consequences of tocopherol engineering may include adjustments in redox and phenylpropanoid metabolism, although such effects are likely to depend on tissue and developmental context.

Flax (*Linum usitatissimum* L.) is a multipurpose oil and fibre crop. Flaxseed oil is characterized by a particularly high content of α-linolenic acid (ALA; 18:3 n-3), which often accounts for more than half of its total fatty acids. This high degree of unsaturation contributes to its nutritional value but also makes the oil particularly susceptible to oxidative deterioration, emphasizing the importance of endogenous lipid-soluble antioxidants such as tocopherols. Flax also exhibits a tissue- and development-dependent tocopherol profile: α-tocopherol predominates in green tissues, whereas γ-tocopherol is the major homologue in mature seeds. During seed maturation, α-tocopherol levels decrease while γ-tocopherol progressively accumulates, resulting in a marked shift in tocopherol composition [10,11].

At the same time, the industrial value of flax depends strongly on the composition and properties of stem tissues. This dual-purpose character makes flax particularly useful for investigating the broader consequences of metabolic engineering, as its PUFA-rich seeds provide a system in which antioxidant protection is especially relevant, whereas its fibre-rich stems allow evaluation of potential effects on cell-wall composition and other structural traits [12,13,14].

Stable genetic transformation of flax has been established for several decades, particularly using *Agrobacterium*-mediated approaches, and has been successfully applied to modify diverse metabolic and structural traits, including phenylpropanoid metabolism and cell-wall-related properties [15,16]. Several endogenous genes involved in tocopherol biosynthesis have previously been characterized at the transcriptional level in flax; however, these studies did not involve targeted genetic manipulation of the pathway. To the best of our knowledge, no previous study has reported direct genetic engineering of the tocopherol biosynthetic pathway in flax. The present study therefore represents the first report of stable tocopherol-pathway engineering in flax through constitutive expression of *Arabidopsis thaliana AtVTE2* and examines its metabolic and developmental consequences.

We investigated whether constitutive *AtVTE2* expression enhanced tocopherol accumulation and whether this modification was accompanied by broader changes in interconnected isoprenoid and antioxidant metabolism, extending from green tissues to mature stems. Given the metabolic links between tocopherol biosynthesis and plastidial isoprenoid metabolism, we hypothesized that constitutive *AtVTE2* expression would increase tocopherol accumulation and be accompanied by coordinated changes in interconnected isoprenoid pathways. We further hypothesized that these metabolic adjustments would be associated with changes in antioxidant and phenylpropanoid metabolism and, in mature stems, with alterations in cell-wall composition. To address these questions, we combined gene-expression analysis with targeted metabolite profiling, antioxidant-capacity measurements, field phenotyping, and biochemical characterization of mature flax straw.

## 2. Results

To evaluate the effects of *AtVTE2* expression on isoprenoid metabolism and antioxidant capacity in flax, transgenic lines were first selected and molecularly verified. The expression of genes associated with tocopherol, methylerythritol phosphate (MEP), mevalonate (MVA), and carotenoid biosynthesis was subsequently analyzed by Real-Time qPCR. Additionally, terpenoids, phytosterols, free and cell wall-associated phenolic compounds, and the antioxidant capacity of methanolic extracts were determined in green tissues of plants grown in vitro.

### 2.1. Selection and Molecular Verification of Transgenic Flax Lines

**Figure 1:**
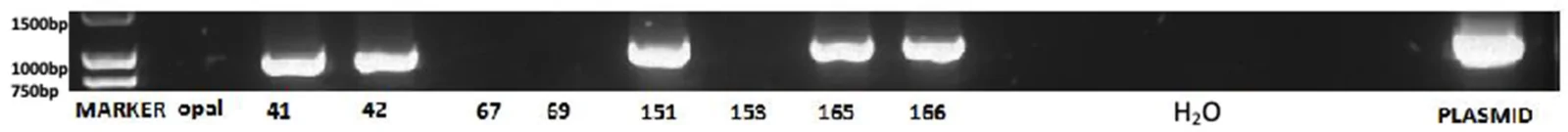
Molecular verification of *AtVTE2* expression in transgenic flax lines. PCR products were amplified from cDNA prepared from green tissues of flax plants grown in vitro. The expected amplicon size was 900 bp. Opal, non-transgenic control; Plasmid, positive control (pBinAR plasmid containing AtVTE2); H_2_O, no-template control; numbers indicate the analyzed transgenic lines.

Genomic DNA isolated from putative transgenic plants was screened for the presence of the transgene, and eight positive lines were selected for further verification. Total RNA was isolated from the selected lines, reverse-transcribed, and the resulting cDNA was analyzed for *AtVTE-2* expression. Three independent lines, designated 42, 151, and 165, expressed the introduced *AtVTE2* gene and were selected for subsequent analyses (Figure 1). Sequencing of the PCR product confirmed that the amplified fragment corresponded to the gene introduced during transformation.

### 2.2. Expression of Genes Associated with Isoprenoid Biosynthesis

#### 2.2.1. Expression of Genes Involved in Tocopherol Biosynthesis

To determine whether *AtVTE2* expression was associated with transcriptional changes in related metabolic pathways, the expression of flax genes involved in tocopherol, MEP, MVA, and carotenoid biosynthesis was analyzed by RT-qPCR.

**Figure 2:**
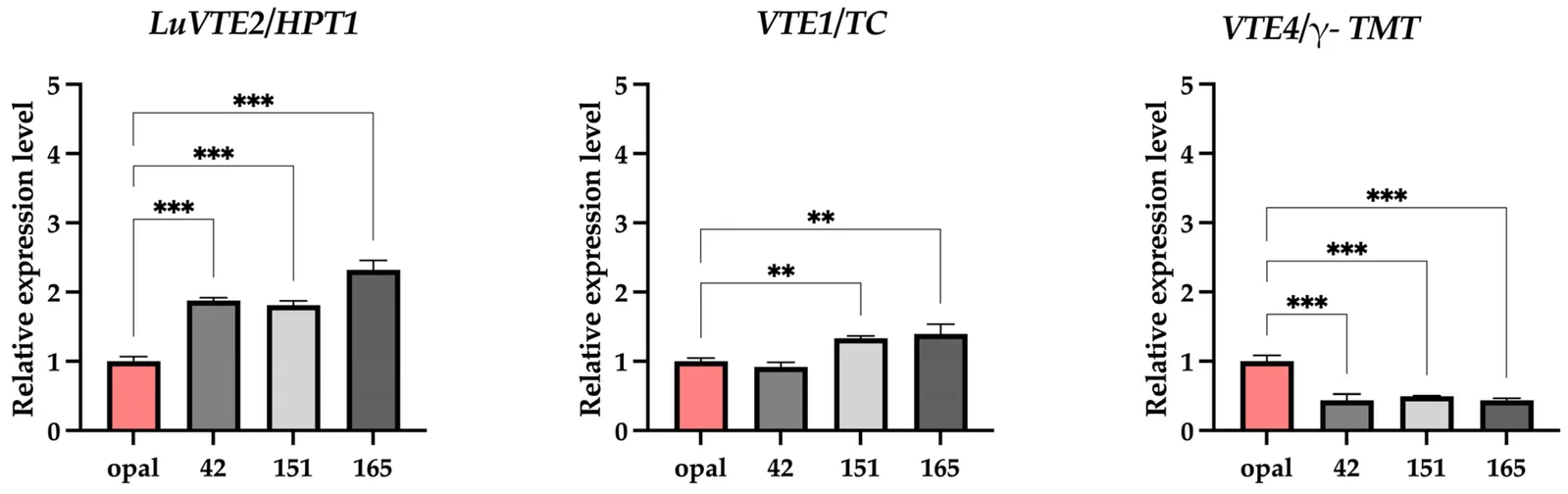
Relative expression of genes encoding enzymes of the flax tocopherol biosynthesis pathway in transgenic flax lines. Transcript levels were determined by RT-qPCR in lines 42, 151, and 165 and expressed relative to the non-transgenic Opal control. Actin was used as the reference gene. *HPT1*, homogentisate phytyltransferase; *TC*, tocopherol cyclase; *γ-TMT*, γ-tocopherol methyltransferase. Data are presented as mean ± SD (n = 3). Statistical significance was assessed using one-way ANOVA: ** *p* < 0.005, and *** *p* < 0.001. Wild-type flax is shown in red, whereas the transgenic lines are shown in greyscale.

Expression of the endogenous flax homogentisate phytyltransferase gene (*VTE2/HPT1*) was significantly increased in all three transgenic lines, by approximately 80% in lines 42 and 151 and by 130% in line 165 relative to the non-transgenic Opal control. Expression of the tocopherol cyclase gene (*VTE1/TC*) was significantly increased by approximately 40% in lines 151 and 165, whereas no significant change was observed in line 42. In contrast, expression of the γ-tocopherol methyltransferase gene (*VTE4/γ-TMT*) was significantly reduced by approximately 50% in all three transgenic lines (Figure 2).

#### 2.2.2. Expression of Genes Involved in MEP and MVA Biosynthesis

The expression of genes encoding enzymes acting at the beginning of the plastidial MEP pathway was also examined.

**Figure 3:**
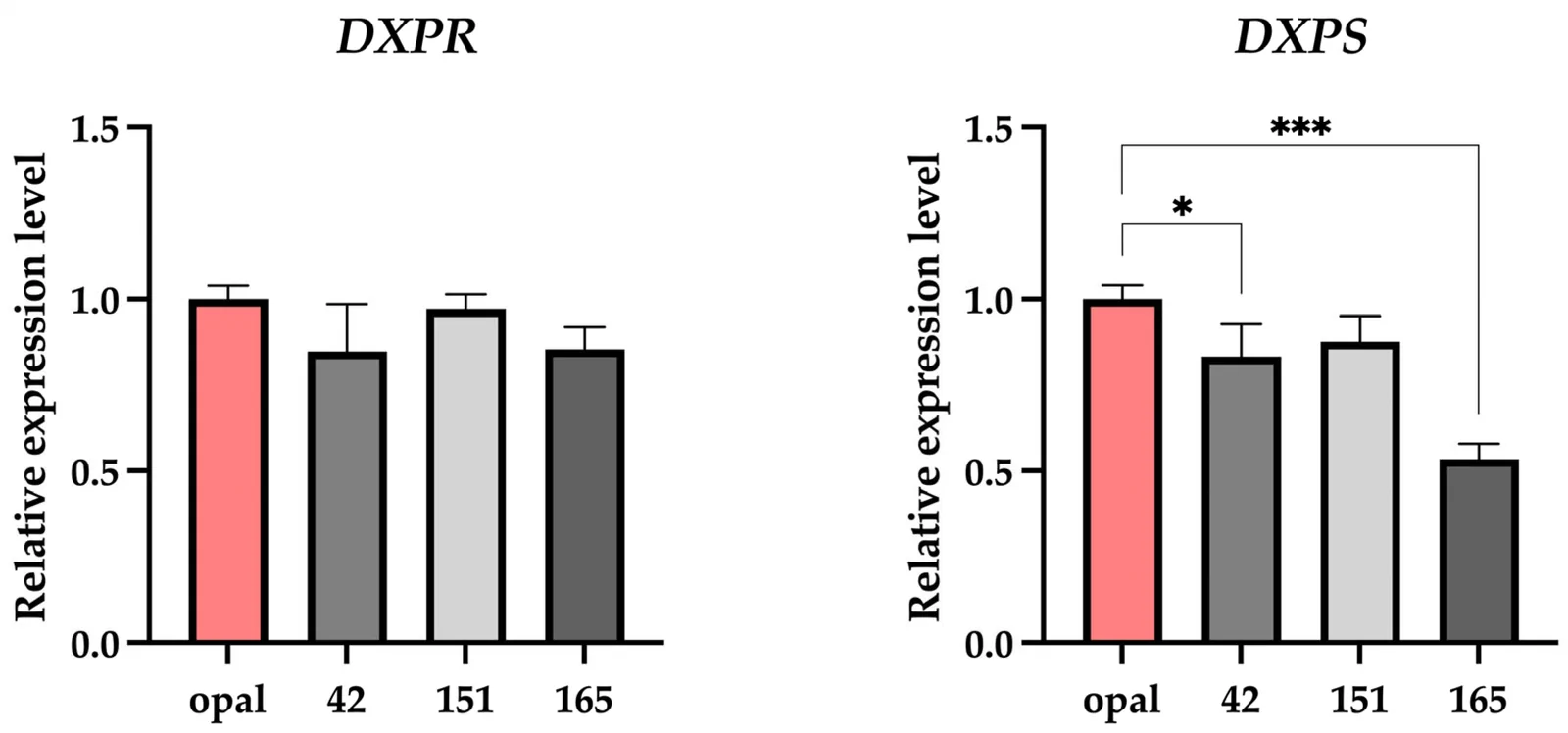
Relative expression of genes encoding enzymes of the plastidial methylerythritol phosphate (MEP) pathway in transgenic flax lines. Transcript levels were determined by RT-qPCR in lines 42, 151, and 165 and expressed relative to the non-transgenic Opal control. Actin was used as the reference gene. *DXPR*, 1-deoxy-D-xylulose 5-phosphate reductoisomerase; *DXPS*, 1-deoxy-D-xylulose 5-phosphate synthase. Data are presented as mean ± SD (n = 3). Statistical significance was assessed using one-way ANOVA; * *p* < 0.05 and *** *p* < 0.001. The non-transgenic Opal control is shown in red, whereas the transgenic lines are shown in greyscale.

Within the plastidial MEP pathway, *DXPS* expression was significantly reduced by approximately 20% in line 42 and by nearly 50% in line 165, whereas no significant change was detected in line 151. *DXPR* expression did not differ significantly from the Opal control in any of the transgenic lines (Figure 3).

**Figure 4:**
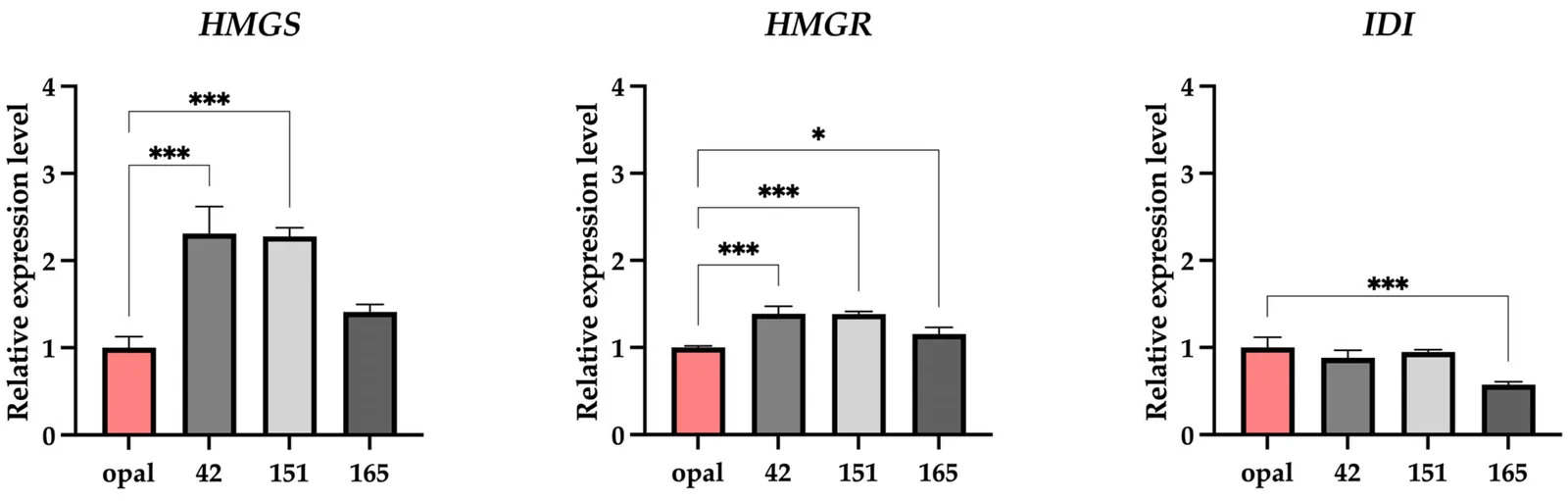
Relative expression of genes encoding enzymes of the cytosolic mevalonate (MVA) pathway in transgenic flax lines. Transcript levels were determined by RT-qPCR in lines 42, 151, and 165 and expressed relative to the non-transgenic Opal control. Actin was used as the reference gene. *HMGS*, 3-hydroxy-3-methylglutaryl-CoA synthase; *HMGR*, 3-hydroxy-3-methylglutaryl-CoA reductase; *IDI*, isopentenyl-diphosphate isomerase. Data are presented as mean ± SD (n = 3). Statistical significance was assessed using one-way ANOVA: * *p* < 0.05 and *** *p* < 0.001. The non-transgenic Opal control is shown in red, whereas the transgenic lines are shown in grey-scale.

Within the cytosolic MVA pathway, *HMGS* expression was significantly increased by approximately 140% in lines 42 and 151, whereas no significant difference was detected in line 165. *HMGR* expression increased significantly in all three transgenic lines, by approximately 40%, 40%, and 20% in lines 42, 151, and 165, respectively. *IDI* expression was significantly reduced by approximately 41% in line 165 but did not differ significantly from the control in lines 42 and 151 (Figure 4).

#### 2.2.3. Expression of Genes Involved in Carotenoid Biosynthesis

**Figure 5:**
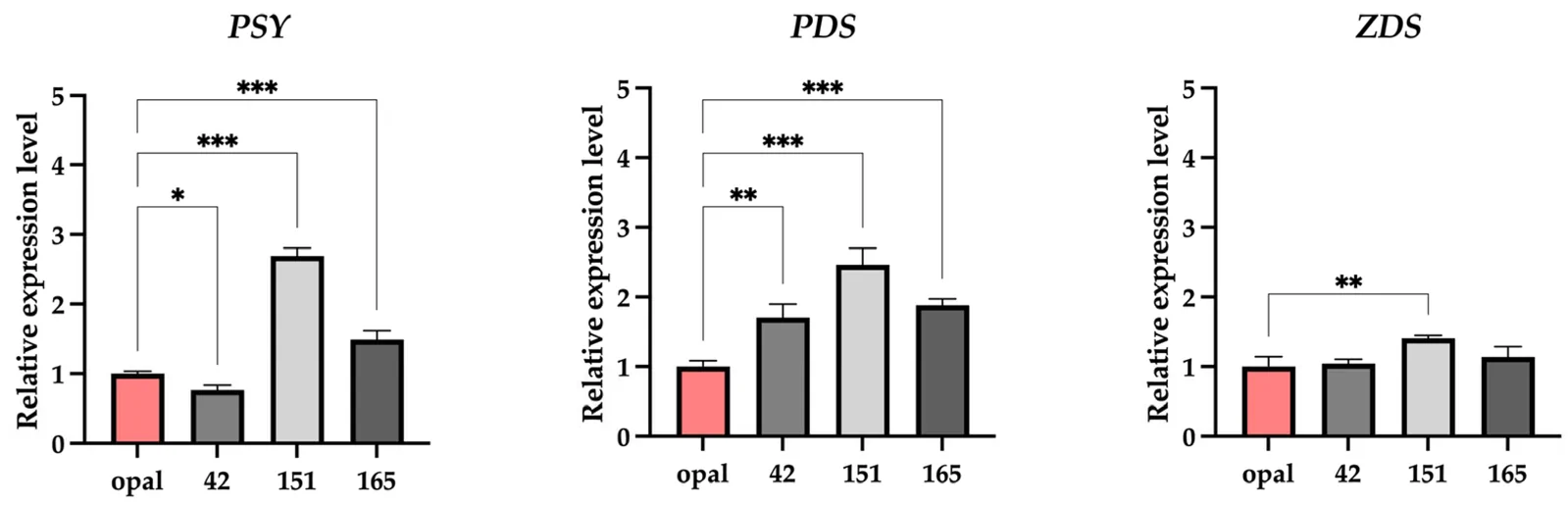
Relative expression of genes encoding enzymes of the carotenoid biosynthesis pathway in transgenic flax lines. Transcript levels were determined by RT-qPCR in lines 42, 151, and 165 and expressed relative to the non-transgenic Opal control. Actin was used as the reference gene. *PSY*, phytoene synthase; *PDS*, phytoene desaturase; *ZDS*, ζ-carotene desaturase. Data are presented as mean ± SD (n = 3). Statistical significance was assessed using one-way ANOVA: * *p* < 0.05, ** *p* < 0.005, and *** *p* < 0.001. The non-transgenic Opal control is shown in red, whereas the transgenic lines are shown in grey-scale.

Phytoene synthase (*PSY*) expression showed a line-dependent response: it was significantly reduced by approximately 25% in line 42 but significantly increased by approximately 170% and 50% in lines 151 and 165, respectively. Phytoene desaturase (*PDS*) expression was significantly elevated in all three transgenic lines, reaching approximately 175%, 250%, and 195% of the control level in lines 42, 151, and 165, respectively. ζ-carotene desaturase (ZDS) expression was significantly increased by approximately 50% in line 151, whereas no significant differences were detected in lines 42 and 165 (Figure 5).

### 2.3. Metabolite Profiles in Green Tissues of Transgenic Flax

#### 2.3.1. Tocopherol, Chlorophyll, and Carotenoid Contents

The primary objective of the genetic modification was to increase tocopherol accumulation in the Opal flax cultivar through the expression of *AtVTE2*, a key gene in the tocopherol biosynthesis pathway. In addition to α-tocopherol, metabolites representing other branches of isoprenoid metabolism were quantified to determine whether the modification was associated with broader changes in terpenoid composition and with metabolites derived from the phenylpropanoid pathway.

Chromatographic analysis of extracts prepared from green tissues of flax plants grown in vitro enabled the identification of seven compounds by comparison of their UV-Vis spectra and retention times with authentic standards. The identified metabolites comprised α-tocopherol; the carotenoids lutein, β-carotene, neoxanthin, and violaxanthin; and chlorophylls a and b.

**Figure 6:**
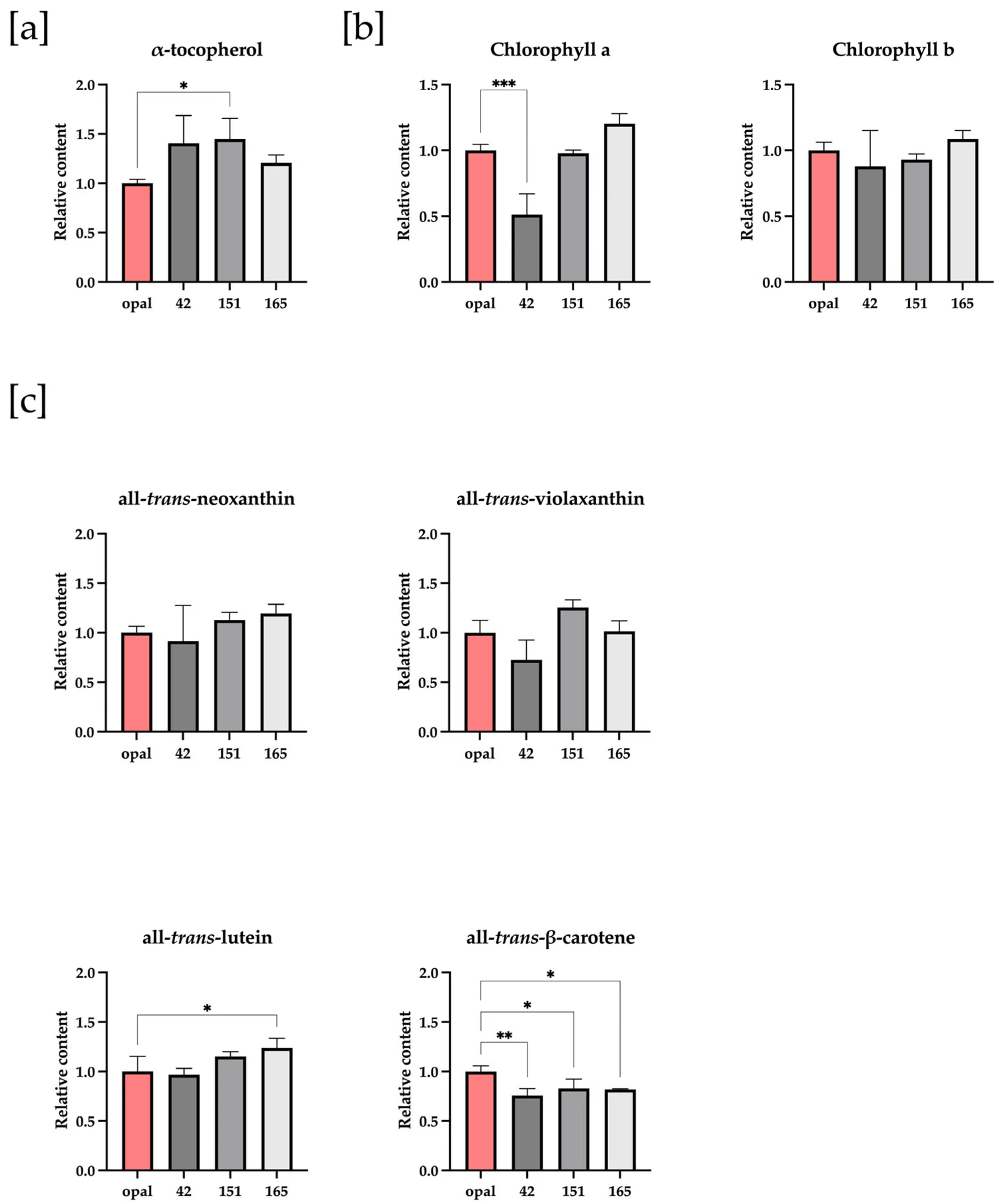
Relative contents of α-tocopherol (**a**), chlorophylls (**b**), and carotenoids (**c**) in green tissues of transgenic flax lines 42, 151, and 165 compared with the non-transgenic Opal control. Compounds were quantified by UPLC-PDA-MS. Data are presented as mean ± SD (n = 3). Statistical significance was assessed using one-way ANOVA: * *p* < 0.05, ** *p* < 0.005, and *** *p* < 0.001. The non-transgenic Opal control is shown in red, whereas the transgenic lines are shown in grey-scale.

α-Tocopherol content was significantly increased in line 151, reaching approximately 145% of the Opal control value. Although the increases observed in lines 42 and 165 did not reach statistical significance, α-tocopherol content was numerically higher than in the control in all three transgenic lines (Figure 6a).

Chlorophyll a content was significantly reduced to approximately 53% of the control value in line 42, whereas no significant differences were detected in lines 151 and 165. Chlorophyll b content did not differ significantly from the control in any of the transgenic lines (Figure 6b). Modification of the tocopherol biosynthesis pathway was also associated with changes in carotenoid accumulation. Among the analyzed carotenoids, lutein content was significantly increased in line 165, reaching approximately 128% of the control value. β-Carotene content was significantly reduced in all three transgenic lines to approximately 79%, 80%, and 82% of the control value in lines 42, 151, and 165, respectively. Neoxanthin and violaxanthin contents did not differ significantly from the Opal control (Figure 6c).

#### 2.3.2. Phytosterol Content

**Figure 7:**
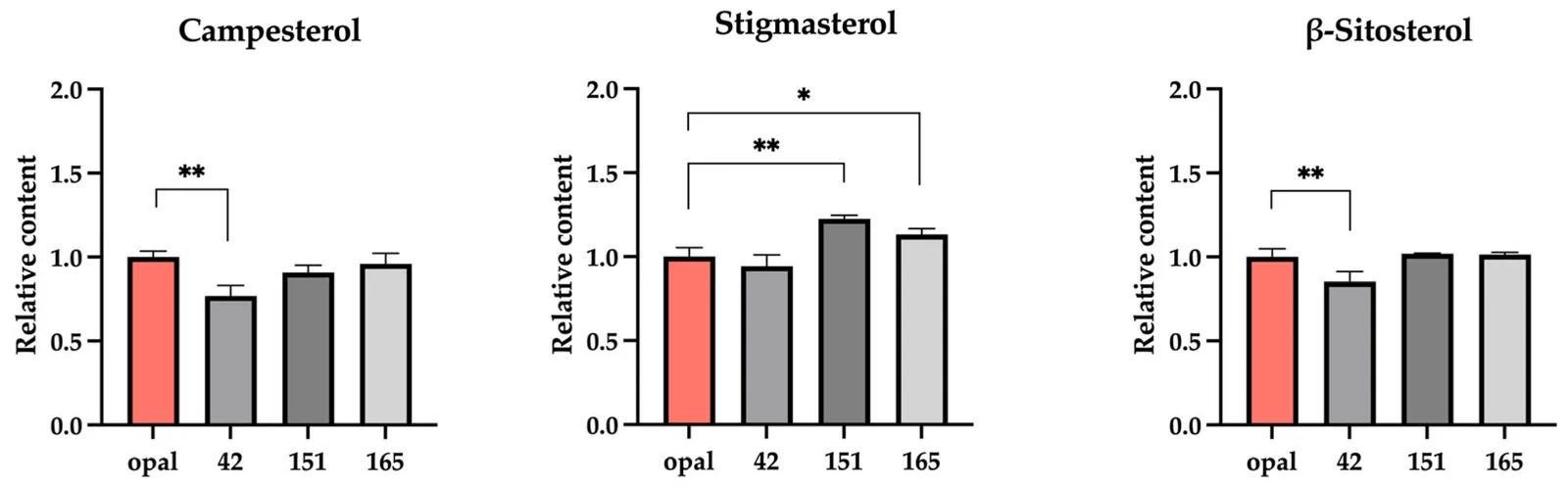
Phytosterol contents in green tissues of transgenic flax lines 42, 151, and 165 compared with the non-transgenic Opal control. Phytosterols were determined by gas chromatography–mass spectrometry (GC-MS) in extracts from plants grown in vitro. Data are presented as mean ± SD (n = 3). Statistical significance was assessed using one-way ANOVA; * *p* < 0.05 and ** *p* < 0.005. The non-transgenic Opal control is shown in red, whereas the transgenic lines are shown in grey-scale.

Phytosterol composition showed compound- and line-specific changes. Campesterol content was significantly reduced to approximately 77% of the control value in line 42, whereas no significant differences were detected in lines 151 and 165. Stigmasterol content was significantly increased to approximately 123% and 113% of the control value in lines 151 and 165, respectively, while line 42 did not differ significantly from the control. β-Sitosterol content was significantly reduced to approximately 85% of the control value in line 42, with no significant differences detected in lines 151 and 165 (Figure 7).

#### 2.3.3. Free Phenolic Compounds

To determine whether the modification of a hydrophobic antioxidant pathway was accompanied by changes in hydrophilic antioxidants, free phenolic compounds were quantified in green tissues of transgenic and control plants grown in vitro using UPLC-PDA-MS.

Seven phenolic compounds were identified in the analyzed extracts. The flavonoid fraction included two luteolin glucosides, isoorientin (luteolin 6-C-glucoside) and orientin (luteolin 8-C-glucoside), and two apigenin glucosides, vitexin (apigenin 8-C-glucoside) and vicenin-2 (apigenin 6,8-di-C-glucoside). Derivatives of chlorogenic, ferulic, and caffeic acids were also detected.

**Figure 8:**
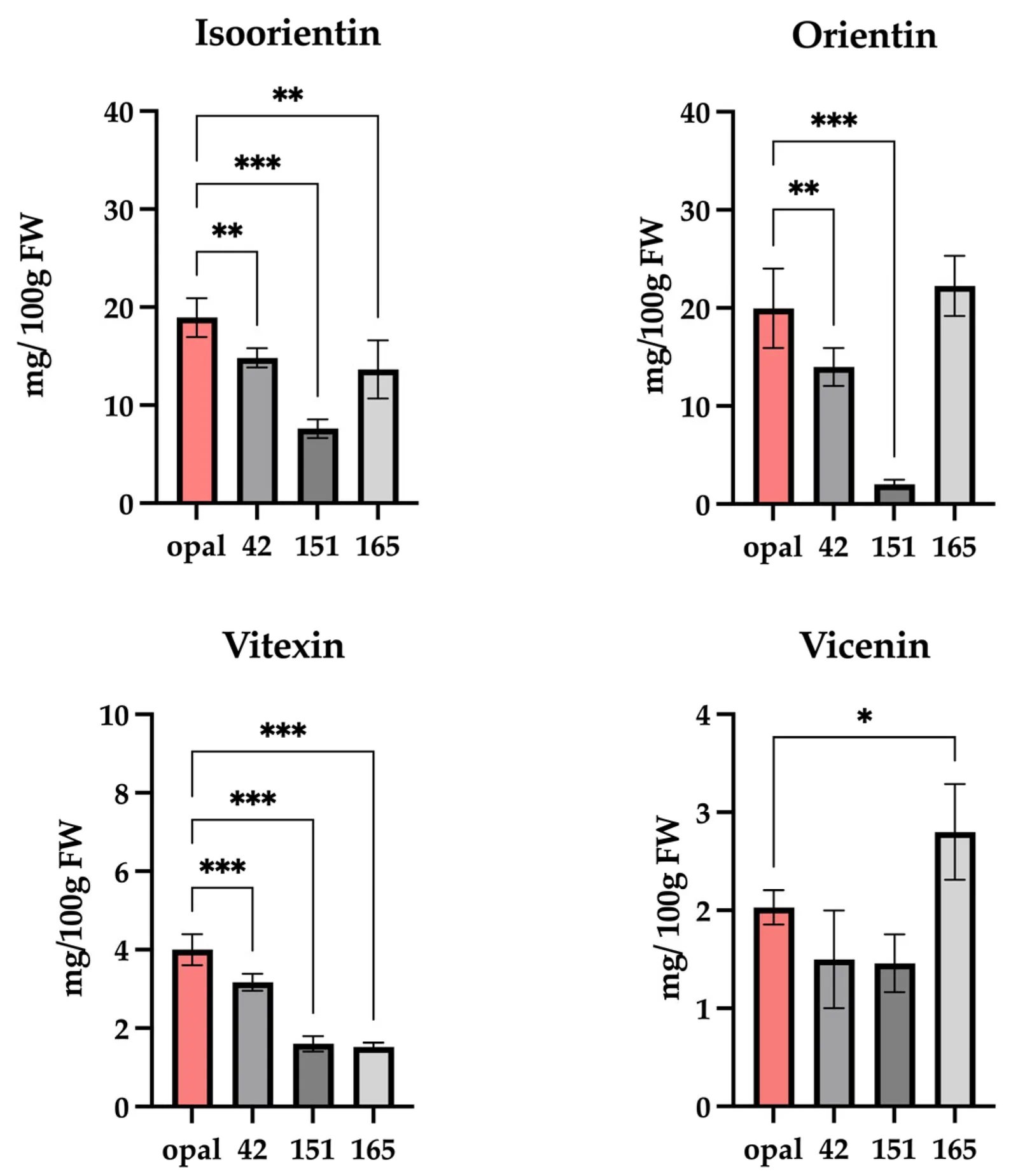
Contents of free flavonoids derived from luteolin (isoorientin and orientin) and apigenin (vitexin and vicenin) in green tissues of transgenic flax lines 42, 151, and 165 compared with the non-transgenic Opal control. Compounds were determined by UPLC-PDA-MS in extracts from plants grown in vitro. Data are presented as mean ± SD (n = 3). Statistical significance was assessed using one-way ANOVA; * *p* < 0.05, ** *p* < 0.005, and *** *p* < 0.001. The non-transgenic Opal control is shown in red, whereas the transgenic lines are shown in grey-scale.

Isoorientin content was significantly reduced in all three transgenic lines, to approximately 78%, 40%, and 72% of the Opal control value in lines 42, 151, and 165, respectively. Orientin content was significantly reduced to approximately 70% and 10% of the control value in lines 42 and 151, respectively, whereas no significant difference was detected in line 165. Vitexin content was also significantly reduced in all three lines, to approximately 79%, 40%, and 38% of the control value, respectively. In contrast, vicenin-2 content was significantly increased to approximately 138% of the control value in line 165, while no significant differences were detected in lines 42 and 151 (Figure 8).

**Figure 9:**
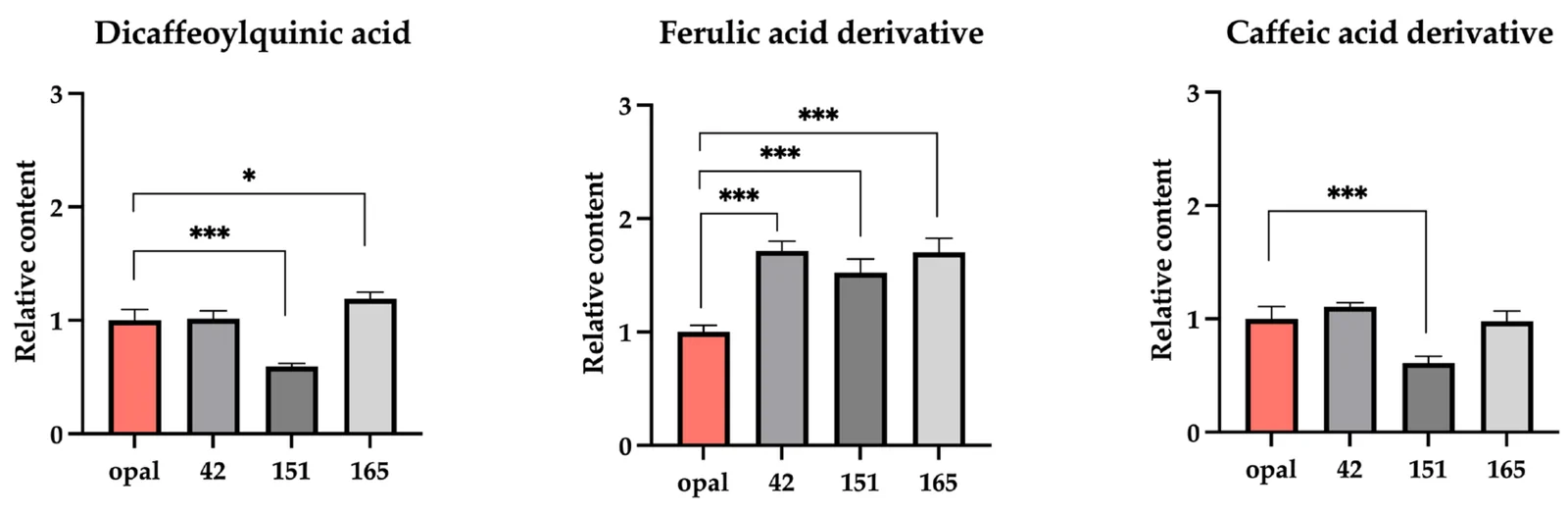
Contents of free phenolic acids and their derivatives in green tissues of transgenic flax lines 42, 151, and 165 compared with the non-transgenic Opal control. Compounds were determined by UPLC-PDA-MS in extracts from plants grown in vitro and are expressed as equivalents of the corresponding phenolic acids. Data are presented as mean ± SD (n = 3). Statistical significance was assessed using one-way ANOVA; * *p* < 0.05 and *** *p* < 0.001. The non-transgenic Opal control is shown in red, whereas the transgenic lines are shown in grey-scale.

The dicaffeoylquinic derivative was significantly reduced to approximately 59% of the control value in line 151 and increased to approximately 119% in line 165, whereas line 42 did not differ significantly from the control. The ferulic acid derivative increased significantly in all three transgenic lines, reaching approximately 171%, 152%, and 170% of the control value in lines 42, 151, and 165, respectively. The caffeic acid derivative was significantly reduced to approximately 61% of the control value in line 151, with no significant differences detected in lines 42 and 165 (Figure 9).

#### 2.3.4. Cell Wall-Associated Phenolic Compounds

**Figure 10:**
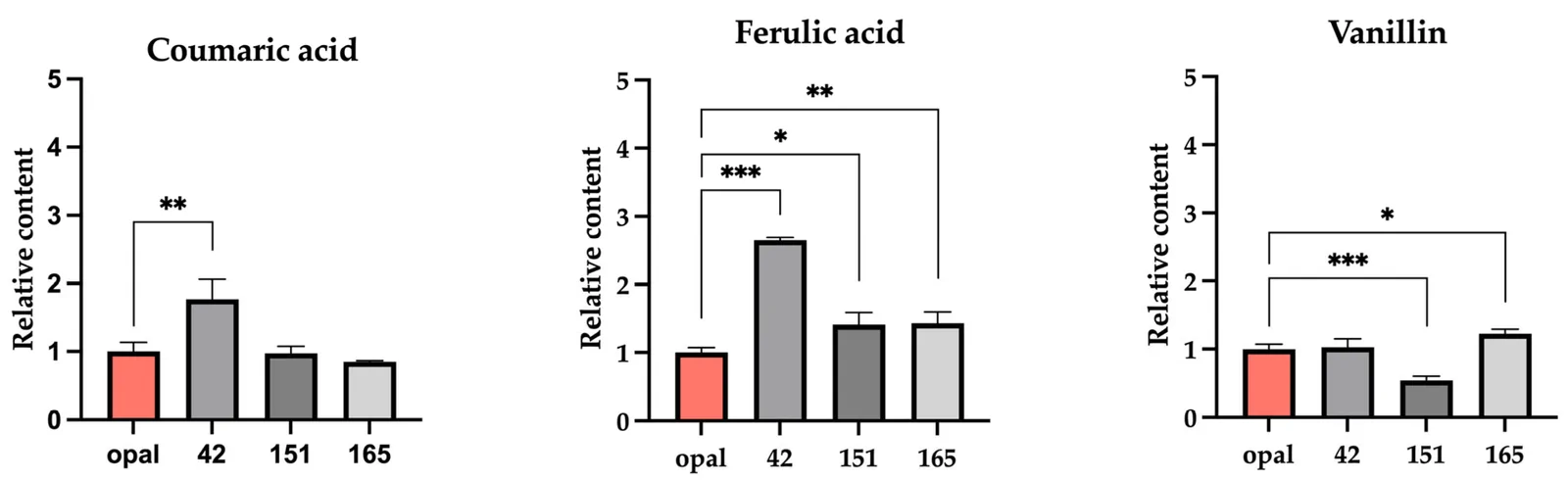
Contents of cell wall-associated phenolic compounds in green tissues of transgenic flax lines 42, 151, and 165 compared with the non-transgenic Opal control. Compounds were determined by UPLC-PDA-MS in extracts from plants grown in vitro. Data are presented as mean ± SD (n = 3). Statistical significance was assessed using one-way ANOVA; * *p* < 0.05, ** *p* < 0.005, and *** *p* < 0.001. The non-transgenic Opal control is shown in red, whereas the transgenic lines are shown in grey-scale.

The p-coumaric acid content was significantly increased to approximately 178% of the Opal control value in line 42, whereas no significant differences were detected in lines 151 and 165. Ferulic acid content increased significantly in all three transgenic lines, reaching approximately 265% of the control value in line 42 and approximately 140% in lines 151 and 165. Vanillin content was significantly reduced by approximately 45% in line 151 and increased to approximately 126% of the control value in line 165, whereas no significant change was observed in line 42 (Figure 10).

### 2.4. Antioxidant Capacity of Methanolic Extracts

Because tocopherols are potent lipid-soluble antioxidants, the increased α-tocopherol content was expected to enhance the antioxidant capacity of transgenic flax tissues. This hypothesis was evaluated using two complementary assays, DPPH and FRAP, in methanolic extracts prepared from green tissues of plants grown in vitro.

**Figure 11:**
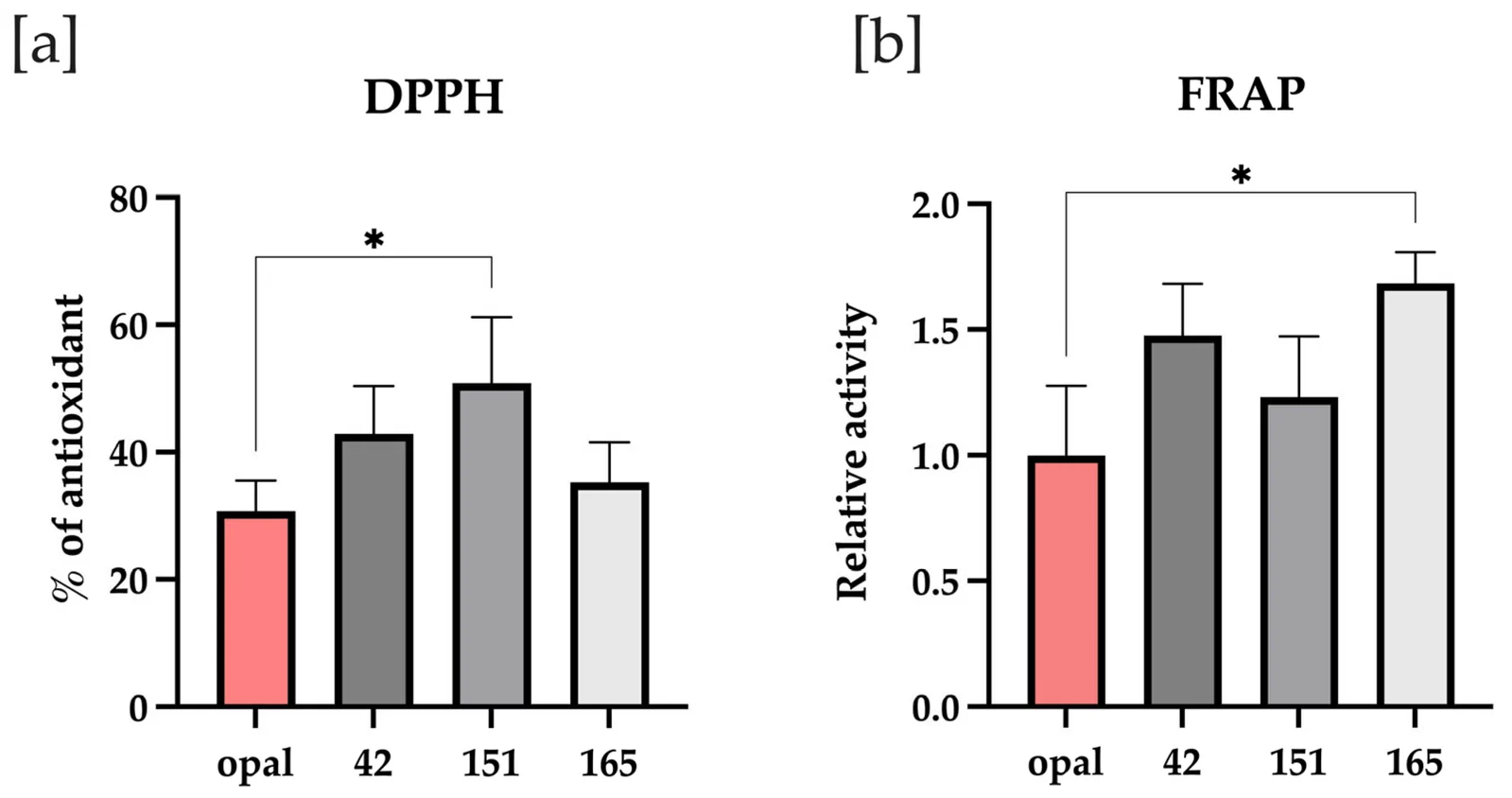
Antioxidant capacity of methanolic extracts prepared from green tissues of transgenic flax lines 42, 151, and 165 and the non-transgenic Opal control, determined using the DPPH radical-scavenging assay (**a**) and the ferric-reducing antioxidant power (FRAP) assay (**b**). Data are presented as mean ± SD (n = 5). Statistical significance was assessed using one-way ANOVA; * *p* < 0.05. The non-transgenic Opal control is shown in red, whereas the transgenic lines are shown in grey-scale.

DPPH radical-scavenging activity was significantly increased in line 151, reaching approximately 166% of the Opal control value (*p* = 0.03), whereas no significant differences were detected in lines 42 and 165 (Figure 11a). FRAP activity was significantly increased in line 165, reaching approximately 168% of the control value (*p* = 0.01), whereas the differences observed in lines 42 and 151 were not statistically significant (Figure 11b).

### 2.5. Effect of AtVTE2 Expression on Plant Phenotype and Productivity

**Figure 12:**
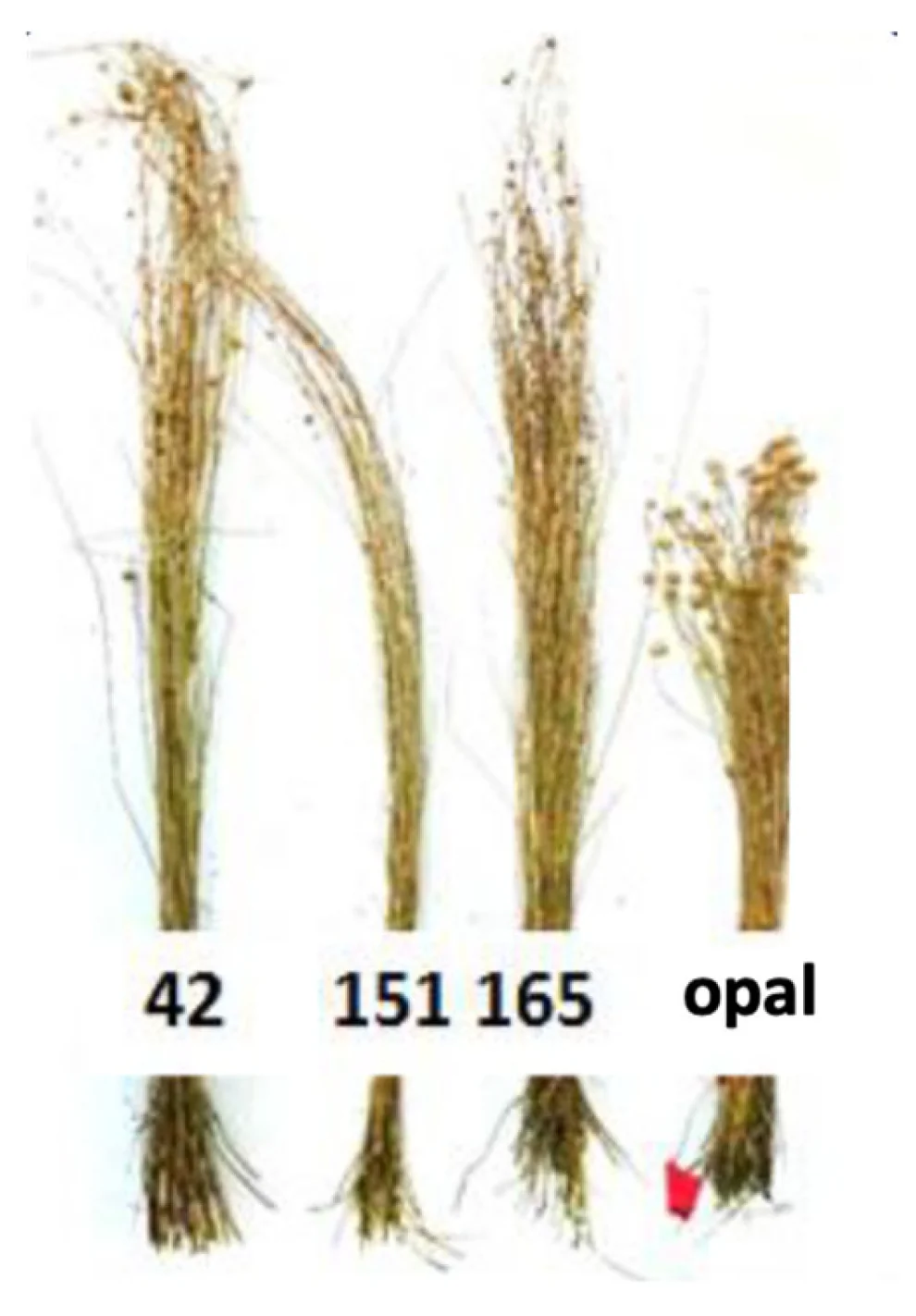
Comparison of the growth habit of transgenic Hom flax lines (42, 151, and 165) and the non-transgenic Opal control cultivated under field conditions.

For field phenotyping, the F1 progeny of transgenic flax lines 42, 151, and 165, together with the non-transgenic Opal control, were grown under field conditions from May to the end of August. No visible differences in flower morphology, leaf shape, or coloration were observed between the transgenic lines and the Opal control. In contrast, all three transgenic lines showed significantly greater plant height (approximately 110 cm compared with 76 cm in Opal) and a significantly higher position of the first branch (47–50 cm compared with 20 cm in the control) (Figure 12). The numbers of flowers and capsules per plant, the number of seeds per capsule, and individual seed weight did not differ significantly from the control. Flowering and seed maturation were delayed by approximately 2–3 weeks in all transgenic lines (Table 1). Thus, the most pronounced phenotypic effects were associated with plant architecture and developmental timing.

The quantitative phenotypic characteristics are summarized in Table 1.

### 2.6. Biochemical Composition of Flax Straw from Field-Grown Transgenic Plants

Following harvest, dried flax straw from field-grown plants was analyzed for selected metabolites and cell-wall components. Comparable mature-straw material was available for transgenic lines 151 and 165 but not for line 42. Therefore, the mature-straw analyses were limited to lines 151 and 165, which were compared with the non-transgenic Opal control.

#### 2.6.1. Phytosterol Content in Flax Straw

**Figure 13:**
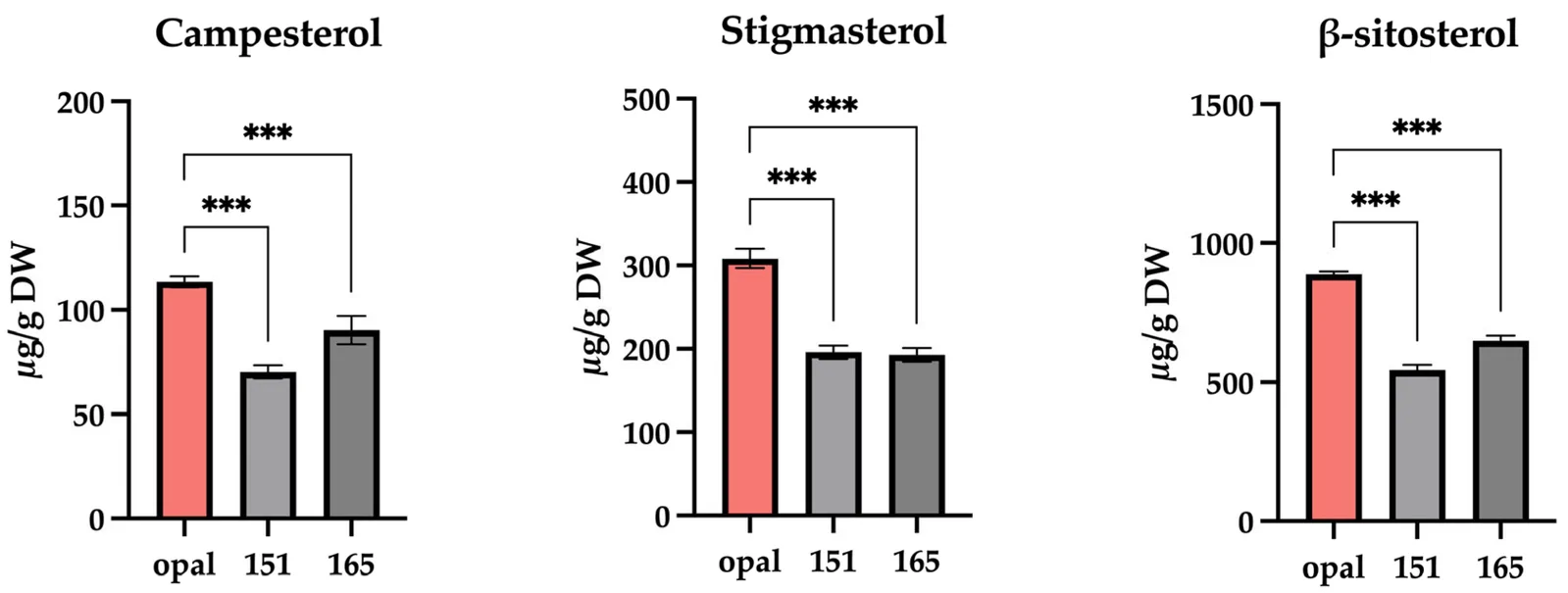
Phytosterol contents in flax straw of transgenic lines 151 and 165 compared with the non-transgenic Opal control. Campesterol, stigmasterol, and β-sitosterol contents are expressed per gram dry weight (DW). Data are presented as mean ± SD (n = 3). Statistical significance was assessed using one-way ANOVA; *** *p* < 0.001. The non-transgenic Opal control is shown in red, whereas the transgenic lines are shown in grey-scale.

The contents of campesterol, stigmasterol, and β-sitosterol were determined to assess whether *AtVTE2* expression affected sterol accumulation in flax straw. All three phytosterols were significantly reduced in both transgenic lines relative to Opal. In line 151, campesterol, stigmasterol, and β-sitosterol contents decreased by 38.7%, 36.4%, and 38.5%, respectively. The corresponding reductions in line 165 were 21.8%, 37.0%, and 26.8% (Figure 13).

#### 2.6.2. Pectin Content

Pectin composition was characterized by separating the water-soluble fraction (WSF), the CDTA-soluble fraction (CSF), and the Na_2_CO_3_-soluble fraction (NSF).

**Figure 14:**
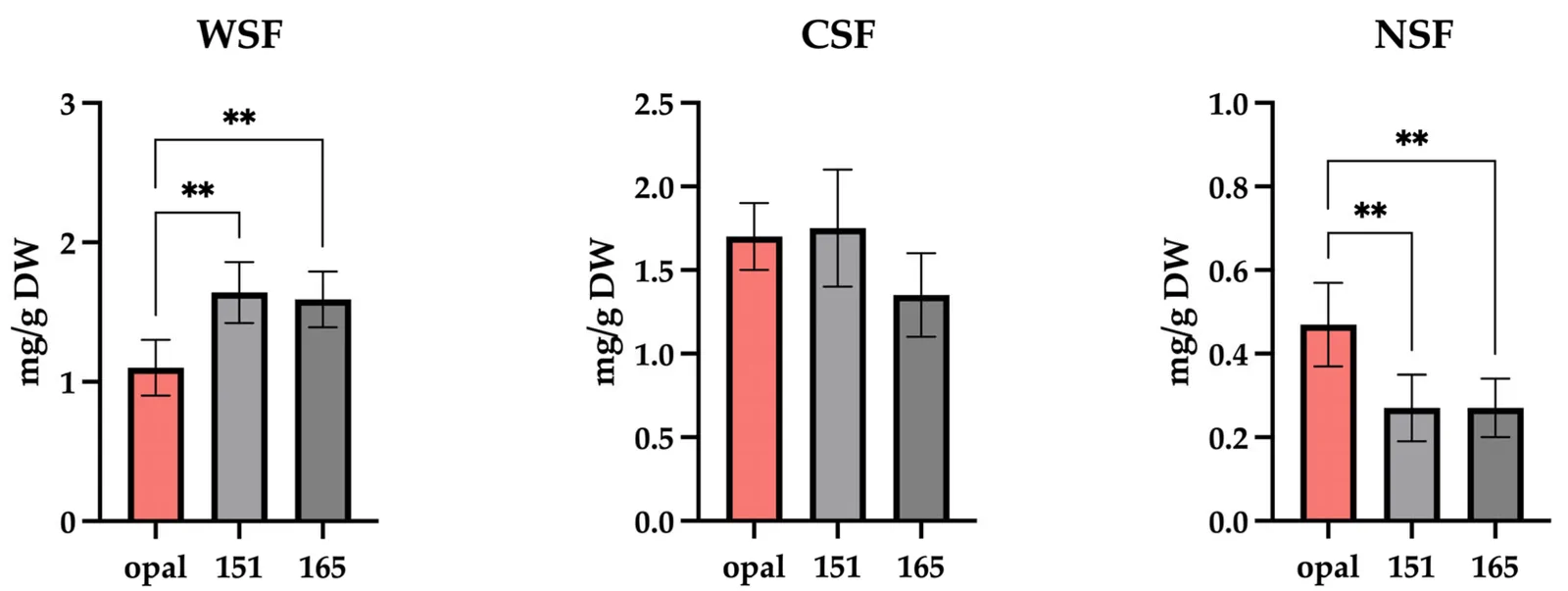
Pectin contents in the water-soluble (WSF), CDTA-soluble (CSF), and Na_2_CO_3_-soluble (NSF) fractions of flax straw from transgenic lines 151 and 165 compared with the non-transgenic Opal control. Contents are expressed mg per gram dry weight (DW). Data are presented as mean ± SD (n = 3). Statistical significance was assessed using one-way ANOVA; ** *p* < 0.005. The non-transgenic Opal control is shown in red, whereas the transgenic lines are shown in grey-scale.

The most pronounced changes in pectin composition were observed in the WSF and NSF fractions. WSF pectin content increased significantly by 49.5% and 44.1% in lines 151 and 165, respectively, whereas NSF pectin content decreased significantly by 38.0% and 42.0%, respectively. No significant differences were detected in the CSF (Figure 14). Total pectin content showed only modest overall differences between the transgenic lines and Opal, with an increase of 12.0% in line 151 and a 2.1% reduction in line 165.

#### 2.6.3. Lignin Content

**Figure 15:**
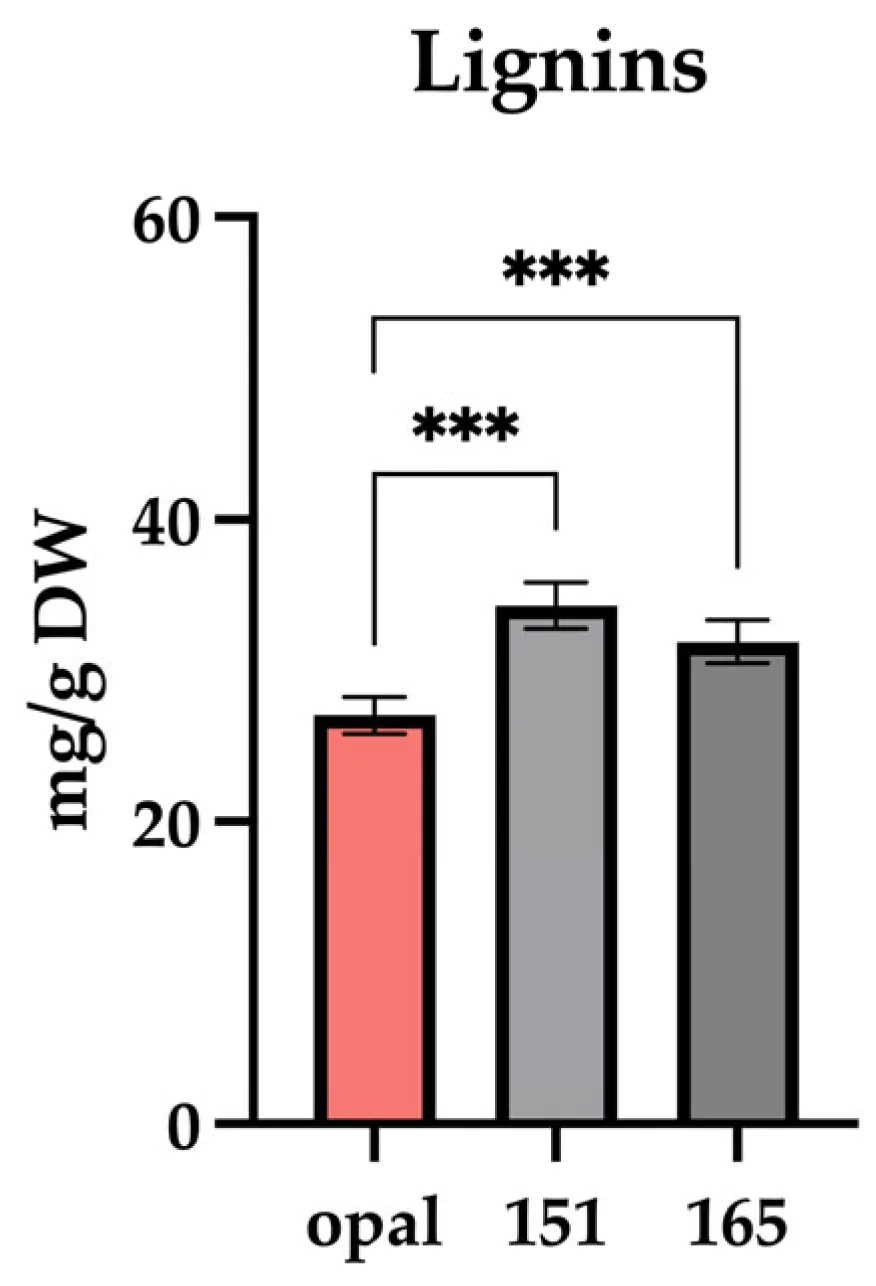
Lignin content in flax straw of transgenic lines 151 and 165 compared with the non-transgenic Opal control. Lignin content is expressed per gram dry weight (DW). Data are presented as mean ± SD (n = 3). Statistical significance was assessed using one-way ANOVA; *** *p* < 0.001. Cellulose content did not differ significantly between transgenic lines 151 and 165 and the Opal control (Figure S1). FT-IR spectra retained the same principal absorption bands across all analyzed samples (Figure S2A), whereas analysis of normalized band areas revealed differences in the relative intensities of selected bands associated with cellulose, pectin, and lignin (Figure S2B). The non-transgenic Opal control is shown in red, whereas the transgenic lines are shown in grey-scale.

Lignin content was quantified to determine whether *AtVTE2* expression affected a major phenolic component of the secondary cell wall. Lignin content was significantly increased relative to Opal, by 27% in line 151 and by 18% in line 165 (Figure 15).

#### 2.6.4. Soluble Flavonoid Content

To determine whether the modification was associated with changes in soluble phenolic metabolites in flax straw, the contents of epigallocatechin, vicenin, isoorientin, an orientin derivative, isovitexin, and vitexin were analyzed.

**Figure 16:**
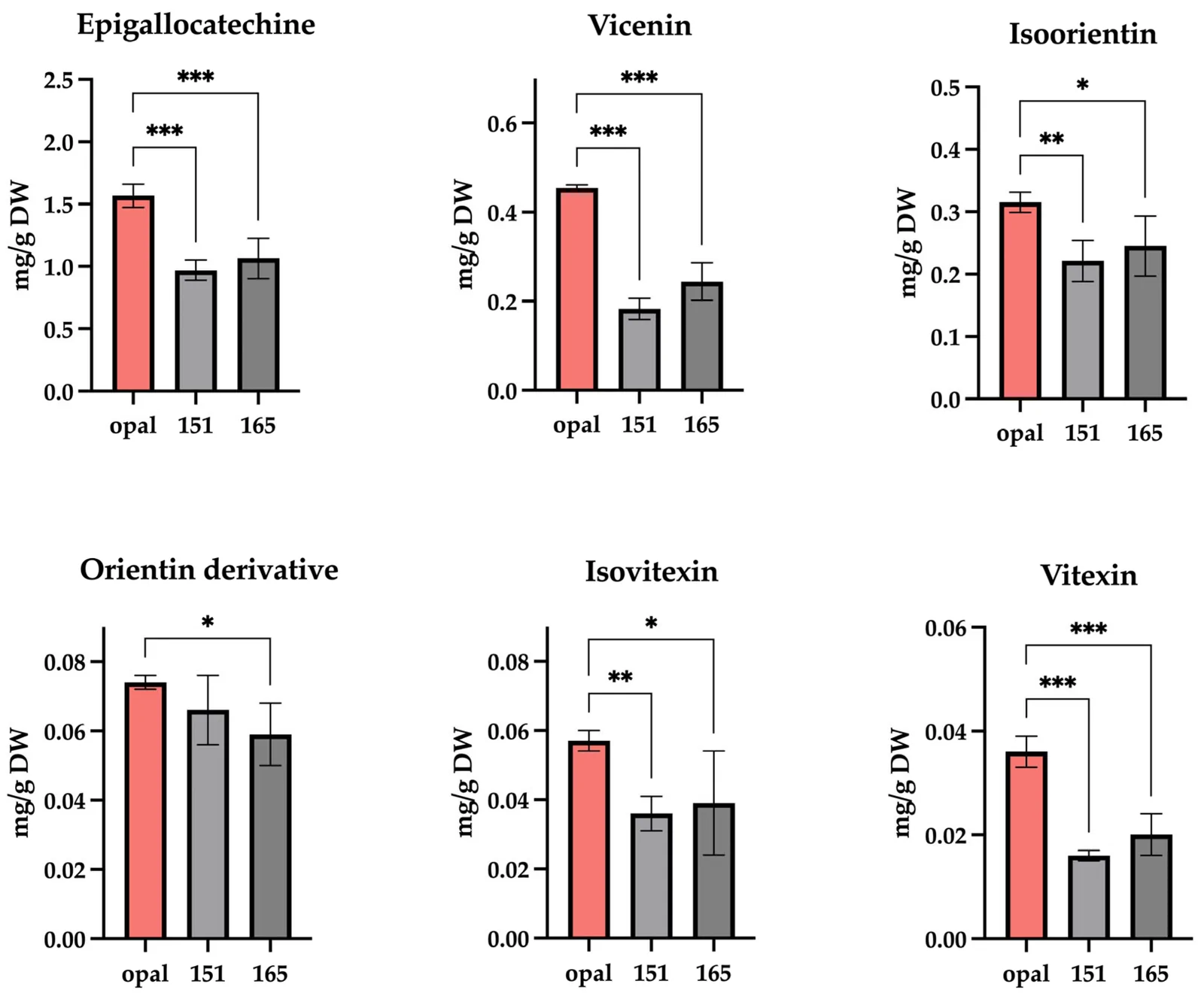
Contents of soluble flavonoids in flax straw of transgenic lines 151 and 165 compared with the non-transgenic Opal control. Components’ contents are expressed per gram dry weight (DW). Data are presented as mean ± SD (n = 3). Statistical significance was assessed using one-way ANOVA; * *p* < 0.05, ** *p* < 0.005, and *** *p* < 0.001. The non-transgenic Opal control is shown in red, whereas the transgenic lines are shown in grey-scale.

In general, both transgenic lines accumulated lower amounts of these compounds than Opal. Epigallocatechin content decreased significantly by 38.3% in line 151 and by 32.1% in line 165. Vicenin content was reduced by 59.6% and 46.4%, respectively, whereas isoorientin decreased by 29.8% in line 151 and by 22.2% in line 165; all of these differences were statistically significant. The orientin derivative showed a significant decrease of approximately 20.3% in line 165. Isovitexin content decreased by 36.8% in line 151 and by 31.6% in line 165, with statistically significant differences in both lines. Vitexin content was also significantly reduced by 58.8% and 51.0% in lines 151 and 165, respectively (Figure 16).

## 3. Discussion

### 3.1. AtVTE2 Expression Was Associated with Increased α-Tocopherol Accumulation and Potential Downstream Methylation Constraints

Constitutive expression of *AtVTE2* was associated with higher α-tocopherol accumulation in all three transgenic lines. Although statistical significance was reached only in line 151, α-tocopherol levels were numerically higher in all three independent transgenic lines, indicating a consistent direction of change that should nevertheless be interpreted cautiously in the two non-significant lines. Published data from other plant species show a much more pronounced effect of *VTE2* overexpression on tocopherol accumulation. For example, overexpression of *AtHPT1/VTE2* increased total tocopherols up to 4.4-fold in *Arabidopsis* leaves, confirming that HPT can strongly control tocopherol pathway flux [3]. Constitutive expression of *AtVTE2* in lettuce produced a more than twofold increase in total tocopherols, predominantly through γ-tocopherol accumulation [17]. Heterologous expression of apple *MdHPT1* in tomato increased α-tocopherol by up to 3.6-fold in leaves but by only 1.7-fold in fruits, illustrating the strong organ dependence of the response [5]. α-Tocopherol is generally the predominant tocopherol in green photosynthetic tissues, particularly in leaves [18]. In flax, the same pattern is observed in young green capsules, whereas γ-tocopherol progressively accumulates during capsule maturation [10]. The increase in endogenous flax *VTE2* and, in two lines, *VTE1* expression was accompanied by lower *VTE4* expression in all three lines. In *Arabidopsis* overexpressing *HPT1*, additional expression of γ-tocopherol methyltransferase redirected the enlarged γ-tocopherol pool toward α-tocopherol, demonstrating that downstream methylation can limit the final α-tocopherol content. The coexistence of increased endogenous *VTE2* expression and reduced *VTE4* expression is not necessarily contradictory. VTE2 acts upstream and may increase pathway input, whereas VTE4 controls a downstream methylation step leading to α-tocopherol [3]. Relative transcript levels of these genes cannot be meaningfully summed because they represent distinct enzymatic steps and do not provide a direct measure of pathway flux. Reduced VTE4 expression may therefore have constrained further α-tocopherol accumulation despite increased upstream pathway activity. However, because minor tocopherol homologues were not quantified, particularly γ-tocopherol, this interpretation cannot be directly verified.

### 3.2. AtVTE2 Expression Was Associated with Opposing Transcriptional Adjustments in the MEP and MVA Pathways

The reduction in *DXPS* expression and induction of *HMGS* and *HMGR* indicate opposing regulation of plastidial and cytosolic isoprenoid metabolism rather than uniform pathway activation. Inhibition of the MVA pathway in *Arabidopsis* transiently reduced sterol accumulation while increasing chlorophylls and carotenoids, showing that perturbation of one compartment can influence products synthesized in the other [6]. Overexpression of *BjHMGS1* in tomato increased both the MVA-derived products squalene and phytosterols and the predominantly MEP-derived α-tocopherol and carotenoids [19]. The induction of *HMGS* and *HMGR* in flax is therefore consistent with compensatory regulation between the pathways. It does not demonstrate direct transfer of cytosolic precursors into tocopherol biosynthesis, because VTE2 uses phytyl diphosphate rather than IPP or DMAPP directly. Because related transcriptional changes were detected across several independent transgenic lines and involved multiple genes of the MEP and MVA pathways, the observed pattern is more consistent with a coordinated response to altered isoprenoid metabolism than with a purely random line-specific effect. Coordination between the plastidial MEP and cytosolic MVA pathways is known to occur at metabolic and regulatory levels, although its underlying mechanisms are complex and include substantial post-transcriptional and post-translational control [20]. Therefore, the present transcript data are consistent with compensatory regulation but do not demonstrate a feedback mechanism directly.

### 3.3. Carotenoid and Chlorophyll Responses Were Branch- and Line-Specific

Induction of several early carotenoid biosynthetic genes was not accompanied by a corresponding increase in carotenoid accumulation. β-Carotene was significantly reduced in all three transgenic lines, whereas lutein increased significantly only in line 165; neoxanthin and violaxanthin did not differ significantly from the control. *HPT1* overexpression in *Arabidopsis* increased tocopherols without producing major changes in chlorophyll, carotenoid or plastoquinone contents [3]. By contrast, in lettuce, *AtVTE2* overexpression reduced chlorophyll content by up to 20%, showing that pigment responses to increased HPT activity differed among species [17]. Reciprocal effects have also been observed after engineering the carotenoid branch, as constitutive *PSY1* expression in tomato increased α- and γ-tocopherol accumulation in mature green fruit [21]. Tocopherols and carotenoids also cooperate in chloroplast photoprotection, as tocopherol-deficient *Arabidopsis* mutants showed severe pigment and lipid oxidation under photooxidative stress [22]. The carotenoid changes in flax may therefore reflect both metabolic partitioning and adjustment of the plastid antioxidant system. Chlorophyll content was maintained in lines 151 and 165 and decreased significantly only in line 42. The apparent increase in chlorophyll a in line 165 was not statistically significant and therefore should not be interpreted as an opposing biological response to that observed in line 42. Notably, line 42 combined a significant reduction in chlorophyll a with reduced *PSY* expression and lower β-carotene accumulation, indicating a more pronounced line-specific perturbation of plastidial metabolism. However, the present data do not distinguish whether this phenotype reflects altered chlorophyll turnover, precursor allocation, or another plastidial regulatory response.

### 3.4. Sterol Composition Did Not Parallel the Transcriptional Response of MVA-Pathway Genes

Induction of *HMGS* and *HMGR* was not accompanied by a general increase in phytosterols. Stigmasterol increased significantly in lines 151 and 165, whereas campesterol decreased significantly in line 42. Overexpression of the sterol C22-desaturases *CYP710A1* and *CYP710A4* in *Arabidopsis* increased stigmasterol at the expense of sitosterol without requiring a general increase in total sterol synthesis [23]. The increase in stigmasterol in green flax tissues may therefore reflect conversion within the sterol pool rather than increased overall MVA pathway flux. In mature field-grown stems, campesterol, stigmasterol and β-sitosterol were reduced in both lines 151 and 165. The opposite stigmasterol responses in green tissues and mature straw suggest that the effect of AtVTE2 on sterol metabolism was tissue- and development-dependent. A similarly organ-specific response was reported in transgenic tobacco, in which constitutive expression of modified AtHMG1 increased seed phytosterols but did not affect their levels in leaves [24]. Developmental regulation of sterol accumulation has also been demonstrated during tobacco and oilseed rape seed maturation [25].

### 3.5. AtVTE2 Expression Was Associated with Phenylpropanoid Remodeling and Line-Specific Changes in Extract Antioxidant Capacity

The aromatic precursor of tocopherols, homogentisate, is derived from tyrosine, whereas phenylpropanoids originate mainly from phenylalanine; both amino acids are produced through the shikimate pathway [26]. Against this biochemical background, the significant reductions in several luteolin- and apigenin-derived C-glycosides, together with the consistent increase in the ferulic acid derivative, indicate selective and line-dependent remodeling of aromatic metabolism. The response was compound-specific rather than uniform, with vicenin-2 showing a significant increase in line 165 despite reductions in several other flavonoids. Although the present data do not demonstrate direct competition for shikimate-derived precursors, they show that *AtVTE2* expression was accompanied by redistribution within phenylpropanoid metabolism. Changes in extract antioxidant capacity were assay- and line-specific. DPPH radical-scavenging activity increased significantly only in line 151, whereas FRAP activity increased significantly only in line 165. Thus, the data do not support a uniform enhancement of antioxidant capacity across the transgenic lines. Instead, they indicate line-dependent changes in the radical-scavenging and ferric-reducing properties of the methanolic extracts. DPPH and FRAP measure different chemical aspects of antioxidant activity and respond differently to individual antioxidant compounds [27]. These assays cannot identify which metabolite or metabolite class was responsible for the observed effects.

Previous studies in Arabidopsis have shown that manipulation of tocopherol metabolism can be accompanied by changes in other antioxidant pools [8]. Such observations make metabolic rebalancing plausible, but the present results do not demonstrate homeostatic compensation between antioxidant pools. Accordingly, the combined tocopherol and phenylpropanoid data are more consistent with selective metabolic redistribution than with maintenance of a fixed total secondary-metabolite or antioxidant pool.

The consistent increase in the ferulic acid derivative and wall-associated ferulic acid points to a reproducible shift in phenylpropanoid allocation associated with *AtVTE2* expression. *Arabidopsis* lines differing in α- and γ-tocopherol composition showed altered phenolic-acid profiles together with changes in pectic epitopes and cell-wall mechanical properties under salinity [9]. The flax profile is therefore consistent with selective and line-dependent remodeling of phenylpropanoid metabolism, with ferulate metabolism representing one of the more consistent responses across the independent transgenic lines.

Field-grown F1 progeny also displayed a consistent architectural phenotype, with increased plant height and a higher position of the first branch, accompanied by delayed flowering and seed maturation, whereas individual seed traits were largely unchanged. These observations indicate that *AtVTE2* expression was associated with developmental effects extending beyond metabolic composition, although the mechanism underlying this phenotype remains unresolved.

### 3.6. The Metabolic Response Extended to the Mature Stem Wall

In mature straw from field-grown lines 151 and 165, lignin content increased while cellulose remained unchanged (Figure S1). Both lines also accumulated more water-soluble pectin and less sodium carbonate-soluble pectin, indicating redistribution of pectin fractions rather than a uniform change in total pectin synthesis. Altered tocopherol composition in *Arabidopsis* was associated with changes in pectin organization and cell-wall mechanics under salinity, supporting a link between tocopherol status and cell-wall properties [9]. A stronger wall phenotype was observed in tocopherol-deficient *Arabidopsis vte2* mutants, which developed abnormal transfer-cell walls and extensive callose deposition under low temperature [28]. Together, these studies show that perturbation of tocopherol metabolism can affect wall deposition and organization under stress conditions, although they do not predict a single direction of change.

The increase in lignin in mature flax straw was accompanied by lower concentrations of several extractable flavonoids, indicating an inverse relationship between the two phenylpropanoid branches. Such reciprocal allocation was demonstrated in HCT-silenced *Arabidopsis*, in which repression of lignin biosynthesis redirected metabolic flux toward flavonoid accumulation [29]. This pattern is consistent with reduced maintenance of soluble phenylpropanoid pools and increased allocation toward wall-associated metabolism, but it does not demonstrate direct conversion of the depleted flavonoids into lignin. Similarly, the coordinated changes in lignin, pectin fractions, and soluble phenylpropanoids indicate remodeling of cell-wall and phenylpropanoid metabolism, but they do not establish that the wall phenotype represents a compensatory mechanism maintaining a fixed total secondary-metabolite or antioxidant pool.

FT-IR spectra retained the same principal absorption bands in transgenic and control straw, indicating preservation of the overall spectral pattern (Figure S2A). However, analysis of normalized band areas revealed differences in the relative intensities of selected cellulose-, pectin-, and lignin-associated bands (Figure S2B). The higher relative intensities of several lignin-associated bands in lines 151 and 165 were consistent with the biochemical increase in lignin content, whereas differences in pectin-associated bands paralleled the redistribution of pectin fractions. Cellulose-associated bands also differed in relative intensity despite the absence of a significant change in total cellulose content, suggesting differences in cellulose organization rather than abundance. Thus, the FT-IR data support structural and compositional remodeling of the mature stem wall without indicating a major change in its overall spectral pattern.

## 4. Methods

### 4.1. Plant Material

Flax (*Linum usitatissimum* L. cv. Opal), a Polish high-linolenic oilseed cultivar characterized by dark-brown seeds, was used as the plant material. Seeds of the high-linolenic flax cultivar Opal were obtained from a seed-production station in Strzelce Opolskie, Poland. The non-transformed Opal cultivar was used as the reference genotype for the generation and subsequent characterization of the transgenic flax lines.

### 4.2. Agrotransformation

Transgenic flax lines were generated using a newly constructed binary vector carrying the *Arabidopsis thaliana* cDNA encoding homogentisate phytyltransferase (*AtVTE2* At2g18950). The coding sequence was introduced in the sense orientation under the control of the constitutive cauliflower mosaic virus 35S (CaMV 35S) promoter and the octopine synthase (OCS) terminator.

Transgenic flax (*Linum usitatissimum* L.) plants were obtained by *Agrobacterium*-mediated transformation of 14-day-old hypocotyl explants from the cultivar Opal according to Lorenc-Kukuła et al. (2005) [15]. Hypocotyls were cut into fragments with a sterile scalpel and co-incubated with 50 µL overnight culture of *Agrobacterium tumefaciens* LBA4404 in liquid Murashige and Skoog (MS) medium supplemented with 2% (*w*/*v*) sucrose. Co-cultivation was conducted for 2 days in darkness at 22–24 °C. The explants were subsequently transferred first to callus-induction medium (7–10 days) and then to shoot-induction medium (7–10 weeks). Following root formation, regenerated plants were subjected to selection and used for further analyses in comparison with non-transformed Opal plants.

#### 4.2.1. Plant Cultivation

Transgenic flax plants were cultivated in vitro in growth chambers (50% relative humidity under a 16 h light/8 h dark photoperiod. During the light period, plants were grown at 21 °C under a light intensity of 23 µmol m^−2^ s^−1^, whereas during the dark period they were maintained at 16 °C) on MS medium. In vitro, Plant Preservative Mixture (PPM™; Plant Cell Technology, Inc., Washington, DC, USA); 300 µL per culture vessel) was added to the medium to prevent bacterial and fungal contamination during tissue culture.

#### 4.2.2. Field Cultivation and Phenotypic Assessment

For field phenotyping, F1 progeny of transgenic flax lines 42, 151, and 165, together with the non-transgenic Opal control, were grown under field conditions from May to the end of August. Ten plants per genotype were evaluated. Plant height and the height of the first branch were measured, and the numbers of flowers and capsules per plant, the number of seeds per capsule, and mean single-seed weight were recorded. Flower morphology, leaf shape, and coloration were assessed visually. Flowering and seed maturation were monitored throughout cultivation and their timing was recorded relative to the Opal control. Quantitative phenotypic data are presented as mean ± SD and were analyzed as described in Section 4.7.

### 4.3. Selection of Transgenic Plants

Putative transgenic plants were initially screened by PCR using primers designed to amplify fragments of the AtVTE2 transgene, approximately 900 bp Genomic DNA isolated from the regenerated plants using the DNeasy kit (Qiagen GmbH, Hilden, Germany) was used as the PCR template. All primers used in the study are listed in Table S1.

Transgene expression was subsequently verified by PCR using cDNA synthesized from RNA isolated from tissue-cultured plants. Total RNA was extracted from plant material using TRIzol Reagent (Invitrogen, Carlsbad, CA, USA) and treated with DNase I according to the manufacturer’s protocol (Thermo Fisher Scientific, Waltham, MA, USA). First-strand cDNA was synthesized using the SuperScript II Reverse Transcriptase system (Invitrogen, Carlsbad, CA, USA) and analyzed with the same *AtVTE2*-specific primer pairs.

Only plants in which the introduced *AtVTE2* sequence was detected were used in further analyses.

### 4.4. Expression Analysis of Genes Involved in the Mevalonate and Non-Mevalonate Terpenoid Biosynthesis Pathways

Total RNA was isolated as describe above. The primers used for quantitative real-time PCR (RT-qPCR) are listed in Table S1. Actin was used as the reference gene for expression normalization.

RT-qPCR was performed using a StepOnePlus™ Real-Time PCR System (Applied Biosystems, Foster City, CA, USA) and the DyNAmo SYBR Green qPCR Kit (Thermo Scientific, Waltham, MA, USA) according to the manufacturers’ instructions. Amplification specificity was verified by melting-curve analysis. Gene expression levels were calculated as relative quantities (RQs) normalized to flax actin gene. Each sample was analyzed in three replicates.

### 4.5. Metabolic Analyses

#### 4.5.1. Analysis of Terpenoid Compounds in Flax Plants

Green plant tissues (stems and leaves) were ground in a ceramic mortar under liquid nitrogen, and 50 mg of material was weighed for each analysis. All extractions were performed in triplicate. The powdered tissue was first extracted with 1 mL of methanol by sonication for 15 min, followed by centrifugation at 16,000 rpm for 10 min at 4 °C. The pellet was then extracted with 1 mL of acetone under the same sonication and centrifugation conditions. A final extraction was performed with 1 mL of petroleum ether. All supernatants were combined and evaporated to dryness under a stream of nitrogen. The residue was dissolved in 200 µL of an acetonitrile–methanol mixture (50:50, *v*/*v*) by sonication for 5 min and analyzed on ACQUITY UPLC system (Waters Corporation, Milford, MA, USA).

##### UPLC–PDA Analysis of Terpenoid Compounds in Flax Plants

The extracts were filtered through a 0.22 µm Acrodisc membrane filter (Gelman Sciences, Ann Arbor, MI, USA) and injected onto an ACQUITY UPLC BEH C18 column (2.1 × 100 mm, 1.7 µm). For the analysis of tocopherol, carotenoids, and chlorophylls, the mobile phase consisted of solvent A (acetonitrile:methanol:water (5:3:2, *v*/*v*)) and solvent B (acetonitrile:methanol (1:1, *v*/*v*)). The following gradient was applied: 1–2 min, 100% A; 10–15 min, 100% B; at 15 min, 100% A. The analytical runs were followed by column washing and re-equilibration. The flow rate was 0.5 mL/min for tocopherol, carotenoid, and chlorophyll analysis.

Absorbance spectra were recorded over the range of 200–500 nm. Tocopherols were quantified at 290 nm, whereas carotenoids and chlorophylls were quantified at compound-specific absorption maxima established using authentic standards. Compounds were identified and quantified by comparison of their retention times and absorption spectra with those of authentic standards.

#### 4.5.2. GC–MS Sterol Analysis in Transgenic Flax Plants

Sterols were extracted and derivatized essentially as described previously by Boba et al. [30] with modifications. Briefly, 200 mg of lyophilized green tissue or 250 mg milled stem was extracted with methanol/chloroform (2:1, *v*/*v*), and the resulting lipid fraction was subjected to alkaline saponification in 2 M KOH in anhydrous methanol using 5α-cholestane as an internal standard. The unsaponifiable fraction was recovered by repeated extraction with hexane and derivatized with BSTFA containing 1% TMCS. The initial extraction time was 20 min and the derivatized sterol fraction was analysed by GC–MS as described below.

The dried unsaponifiable fraction was derivatized by adding 100 µL of N,O-bis(trimethylsilyl)trifluoroacetamide (BSTFA) containing 1% trimethylchlorosilane (TMCS). The samples were mixed for 2 min and incubated at 70 °C for 45 min. After derivatization, the solvent was evaporated under nitrogen, and the residue was dissolved in 200 µL of hexane.

##### Determination of Sterol Content in Flax Plants and Seeds by GC–MS

Sterols were analyzed using a Clarus 680 gas chromatograph coupled to a Clarus SQ 8T mass spectrometer (PerkinElmer, Waltham, MA, USA) and equipped with an HP-5 capillary column (30 m × 0.32 mm × 0.25 µm; Agilent Technologies, Santa Clara, CA, USA), according to the sample-preparation procedure described by Shukla et al. [31]. The injection volume was 0.5 µL for seed samples and 3 µL for green-tissue samples. Helium was used as the carrier gas at a flow rate of 1 mL/min.

The oven temperature was initially maintained at 250 °C for 5 min, increased to 290 °C at a rate of 5 °C/min, and then maintained at 290 °C for 14 min. The total analysis time was 30 min. Mass spectra were recorded in electron-ionization mode at 70 eV over an m/z range of 200–600, with a scan time of 1 s and an ion-source temperature of 220 °C. Data were processed using TurboMass software, version 6.1.0.

Compounds for which authentic standards were available were identified by comparison of retention times and mass spectra with those of the corresponding standards. Mass spectra of peaks lacking authentic standards were compared with the NIST MS Search 2.2 library. When no satisfactory library match was obtained, identification was supported by comparison with published sterol spectra [32,33]. Chromatographic separation and compound identification were performed concurrently during GC–MS analysis.

### 4.6. Analysis of Phenolic Compounds in Flax Plants

Extraction of free and cell-wall-bound phenolic compounds was based on previously described procedures for flax [34,35], with modifications for sample mass and extraction volume. Green tissues (stems and leaves) were ground under liquid nitrogen and 20 mg was used per analysis, whereas dry stems (flax straw) were milled and 50 mg was used. All extractions were performed in triplicate, and reagent volumes were increased proportionally for straw samples.

Briefly, free phenolic compounds were extracted three times with methanol by sonication for 15 min at room temperature. The combined supernatants were evaporated to dryness under vacuum and dissolved in 200 µL of methanol. The residual pellet was then subjected to alkaline hydrolysis with 2 M NaOH for 48 h at 37 °C to release cell-wall-bound phenolics. After centrifugation, the supernatant was adjusted to pH 3.0 with HCl and extracted three times with ethyl acetate. The combined ethyl acetate fractions were evaporated to dryness and dissolved in 200 µL of methanol.

Both non-hydrolyzed and hydrolyzed extracts were analyzed by ultra-performance liquid chromatography coupled with photodiode-array and mass-spectrometric detection (UPLC–PDA–MS).

#### 4.6.1. UPLC–PDA–MS Analysis of Hydrophilic Compounds in Flax Plants

Phenolic compounds present in the extracts were analyzed using a Waters AC-QUITY UPLC system equipped with a 2996 photodiode-array detector and coupled to a Waters Xevo QTof mass spectrometer (Waters Corporation, Milford, MA, USA). Separation was performed on an ACQUITY UPLC BEH C18 column (2.1 × 100 mm, 1.7 µm). The mobile phase consisted of solvent A (0.1% formic acid, pH 3.0) and solvent B (acetonitrile). The following gradient was applied: 0–1 min, 95% A/5% B; 2–12 min, a linear gradient to 75% A/25% B; 13–15 min, a linear gradient to 0% A/100% B; and at 15 min, return to 95% A/5% B. The flow rate was 0.4 mL/min. The analytical run was followed by column washing and re-equilibration.

Mass spectra were recorded for 13 min in positive electrospray-ionization mode over an m/z range of 50–800. The operating parameters were as follows: nitrogen flow, 800 L/h; source temperature, 70 °C; desolvation temperature, 400 °C; capillary voltage, 3.50 kV; sampling-cone voltage, 30 V; cone-voltage ramp, 40–60 V; and scan time, 0.2 s. UV chromatograms were monitored at 280 nm. Compounds were identified and quan-tified by comparison of their retention times and UV and mass spectra with those of authentic standards.

#### 4.6.2. Cell Wall Isolation and Fractionation

Cell wall material was isolated from dried flax straw according to Manganaris et al. [36] with modifications by Wojtasik et al. [37]. Briefly, 200 mg of finely ground dry straw was sequentially extracted with 96% ethanol (2 mL; 30 min at 40 °C under sonication, followed by 20 min at 100 °C), 80% ethanol (5 mL; 20 min at 80 °C), methanol/chloroform (1:1, *v*/*v*; 5 mL; 1 h at 40 °C with agitation), and acetone (5 mL). The ethanol extracts were centrifuged at 5000 rpm for 5 min, the methanol/chloroform extract at 7000 rpm for 5 min, and the acetone wash at 1000 rpm for 10 min; supernatants were removed after each step. The residue was dried overnight at 37 °C to obtain the alcohol-insoluble residue (AIR).

For cell wall fractionation, 100 mg of AIR was extracted sequentially with water, 50 mM CDTA (pH 6.5), and 50 mM Na_2_CO_3_ containing 20 mM NaBH_4_ to obtain the water-soluble (WSF), CDTA-soluble (CSF), and Na_2_CO_3_-soluble (NSF) pectin fractions, respectively. Each fraction was extracted with 15 mL of the corresponding solution for 12 h, followed by centrifugation at 6000 rpm for 10 min at 4 °C. The residue was washed once with 3 mL of the same extraction solution, and the supernatants were combined. CSF and NSF extractions were performed at 4 °C with agitation. The CSF and NSF fractions were dialyzed against water for 12 h using SERVAPOR dialysis membranes, frozen at −80 °C, and lyophilized.

##### Determination of Pectin Content

Total pectin content was estimated as the sum of galacturonic acid equivalents determined in the WSF, CSF, and NSF fractions.

#### 4.6.3. Determination of Lignin Content

Lignin content was determined using the acetyl bromide method of Iiyama and Wallis [38] with modifications. Briefly, 20 mg of ground flax straw was incubated with 2 mL of deionized water at 40 °C for 30 min and centrifuged at 14,000 rpm for 10 min. The pellet was sequentially washed with 1 mL each of water, ethanol, acetone, and diethyl ether and dried overnight at room temperature.

The dried material was incubated with 2.5 mL of 25% acetyl bromide in acetic acid at 50 °C for 2 h. After cooling, the digest was transferred to 10 mL of 2 M NaOH, and the reaction vial was rinsed with 12 mL of acetic acid, which was added to the same tube. Samples were left overnight, and absorbance was measured at 280 nm in quartz cuvettes. Lignin content was quantified using a coniferyl alcohol calibration curve.

### 4.7. Determination of Antioxidant Activity in Transgenic Flax

Extracts for antioxidant-capacity measurements were prepared according to Mierziak et al. [34] with modifications. Briefly, 200 mg of green tissue (stems and leaves from tissue-cultured transgenic flax plants and the Opal control) was ground in liquid nitrogen. The material was extracted three times for 15 min in an ultrasonic bath using methanol or 80% (*v*/*v*) methanol, as appropriate for the analyzed extract fraction. After centrifugation at 14,000 rpm for 10 min, the combined supernatants were evaporated to dryness under nitrogen and dissolved in 500 µL of methanol.

#### 4.7.1. Determination of Antioxidant Activity by the DPPH Method

The radical-scavenging activity of the methanolic extracts was determined using the DPPH assay as described by Mierziak et al. [34]. A 6 µL aliquot of a threefold-diluted extract was added to 200 µL of freshly prepared 0.1 mM DPPH solution in methanol. The samples were incubated for 45 min in darkness at room temperature, and absorbance was measured at 515 nm. A control sample containing 6 µL of methanol instead of the extract was analyzed in parallel. DPPH radical-scavenging activity was calculated as [1 − (A_sample/A_control)] × 100%, where A_sample is the absorbance of the sample and A_control is the absorbance of the control.

#### 4.7.2. Determination of Antioxidant Activity by the FRAP Method

Ferric-reducing antioxidant power was determined using the FRAP assay described by Benzie and Strain [39] and previously applied to flax extracts by Mierziak et al. [34]. The assay is based on the reduction of the ferric tripyridyltriazine complex (Fe(III)–TPTZ) to Fe(II)–TPTZ under acidic conditions.

Six microlitres of a threefold-diluted methanolic plant extract was added to 200 µL of freshly prepared FRAP reagent (300 mM acetate buffer, 20 mM FeCl_3_, and 10 mM TPTZ mixed at a ratio of 10:1:1, *v*/*v*/*v*). Pure methanol was used as the blank. Absorbance at 595 nm was measured immediately after mixing and again after 4 min of incubation at 37 °C. Antioxidant capacity was calculated from the change in absorbance and quantified using a Trolox calibration curve.

Methods for cellulose determination and FT-IR analysis are provided in the Supplementary Materials.

### 4.8. Statistical Analysis

All experiments were performed using at least three biological replicates. Results are presented as mean values ± standard deviation (SD). Statistical analyses were conducted using GraphPad Prism 10 (GraphPad Software, San Diego, CA, USA). Differences among groups were evaluated by one-way analysis of variance (ANOVA), followed by Dunnett’s multiple-comparisons test. Differences were considered statistically significant at * *p* < 0.05, ** *p* < 0.005, and *** *p* < 0.001.

## 5. Conclusions

Constitutive *AtVTE2* expression increased α-tocopherol in green flax tissues, but its metabolic effects extended beyond the tocopherol pathway. The modification was associated with opposing transcriptional adjustments in MEP- and MVA-pathway genes, selective changes in carotenoids and sterols, and line-dependent remodelling of soluble C-glycosylated flavones accompanied by consistent increases in the ferulic acid derivative and wall-associated ferulic acid. In mature straw, *AtVTE2* expression was associated with increased lignification and redistribution of pectin fractions without a significant change in cellulose content, while FT-IR analysis indicated preservation of the overall spectral pattern together with changes in selected cell-wall-associated bands.

## Figures and Tables

**Table 1:** Effect of genetic modification on the phenotype of aerial parts of transgenic flax lines (42, 151, and 165) and the non-transgenic Opal control. Data are presented as mean ± SD (n = 10). Statistically significant difference compared with the Opal control (* *p* < 0.05). na—not affected.

	Opal	42	151	165
Number of plants	10	10	10	10
Mean number of flowers/plant	15.00 ± 1.20	10.20 ± 4.00	9.00 ± 2.00	8.70 ± 2.00
Mean number of capsules/plant	12.00 ± 1.00	8.50 ± 1.00	7.50 ± 1.50	7.00 ± 3.00
Mean number of seeds/capsule	8.80 ± 1.50	8.00 ± 2.00	7.00 ± 2.00	9.00 ± 1.00
Mean weight of a single seed (mg)	5.12 ± 0.20	5.16 ± 0.10	5.13 ± 0.20	5.10 ± 0.30
Flower size	na	na	na	na
Plant height (cm)	76.00 ± 3.00	110.00 ± 10.00 *	110.00 ± 10.00 *	110.00 ± 10.00 *
Height to first branch (cm)	20.00 ± 2.00	50.00 ± 2.00 *	49.00 ± 3.00*	47.00 ± 4.00 *
Flowering	normal	2–3 weeks later		
Seed maturation	normal	2–3 weeks later		

## Data Availability

The raw data supporting the conclusions of this article will be made available by the authors on request.

## Supplementary Materials

The following supporting information can be downloaded at: https://www.mdpi.com/article/10.3390/ijms27188295/s1.

## Abbreviations

AIR	alcohol-insoluble residue
ANOVA	analysis of variance
AtVTE2	Arabidopsis thaliana VTE2, encoding homogentisate phytyltransferase
BSTFA	N,O-bis(trimethylsilyl)trifluoroacetamide
CaMV 35S	cauliflower mosaic virus 35S promoter
CDTA	trans-1,2-diaminocyclohexane-N,N,N′,N′-tetraacetic acid
CSF	CDTA-soluble fraction
DMAPP	dimethylallyl diphosphate
DPPH	2,2-diphenyl-1-picrylhydrazyl
DXPR	1-deoxy-D-xylulose 5-phosphate reductoisomerase
DXPS	1-deoxy-D-xylulose 5-phosphate synthase
FRAP	Ferric-reducing antioxidant power
FT-IR	Fourier-transform infrared spectroscopy
GC–MS	gas chromatography–mass spectrometry
HMGR	3-hydroxy-3-methylglutaryl-CoA reductase
HMGS	3-hydroxy-3-methylglutaryl-CoA synthase
HPT	homogentisate phytyltransferase
HPT1	homogentisate phytyltransferase 1
IDI	isopentenyl-diphosphate isomerase
IPP	isopentenyl diphosphate
MEP	2-C-methyl-D-erythritol 4-phosphate pathway
MS	Murashige and Skoog medium
MVA	mevalonate pathway
NSF	Na_2_CO_3_-soluble fraction
OCS	octopine synthase terminator
PDA	photodiode-array detector
PDS	phytoene desaturase
PPM	Plant Preservative Mixture
PSY	phytoene synthase
RQ	relative quantity
RT-qPCR	reverse-transcription quantitative real-time PCR
SD	standard deviation
TC	tocopherol cyclase
TMCS	trimethylchlorosilane
TPTZ	2,4,6-tripyridyl-s-triazine
UPLC	ultra-performance liquid chromatography
UPLC–PDA	ultra-performance liquid chromatography coupled with photodiode-array detection
UPLC–PDA–MS	ultra-performance liquid chromatography coupled with photodiode-array and mass-spectrometric detection
VTE1	tocopherol cyclase
VTE2	homogentisate phytyltransferase
VTE4/γ-TMT	γ-tocopherol methyltransferase
WSF	water-soluble fraction
ZDS	ζ-carotene desaturase

## References

[1] Cela J., Tweed J.K.S., Sivakumaran A., Lee M.R.F., Mur L.A.J., Munné-Bosch S. (2018). An altered tocopherol composition in chloroplasts reduces plant resistance to Botrytis cinerea. Plant Physiol. Biochem..

[2] Munné-Bosch S. (2005). The role of alpha-tocopherol in plant stress tolerance. J. Plant Physiol..

[3] Collakova E., DellaPenna D. (2003). Homogentisate phytyltransferase activity is limiting for tocopherol biosynthesis in Arabidopsis. Plant Physiol..

[4] Sattler S.E., Gilliland L.U., Magallanes-Lundback M., Pollard M., DellaPenna D. (2004). Vitamin E is essential for seed longevity and for preventing lipid peroxidation during germination. Plant Cell.

[5] Seo Y.S., Kim S.J., Harn C.H., Kim W.T. (2011). Ectopic expression of apple fruit homogentisate phytyltransferase gene (MdHPT1) increases tocopherol in transgenic tomato (*Solanum lycopersicum* cv. Micro-Tom) leaves and fruits. Phytochemistry.

[6] Laule O., Fürholz A., Chang H.-S., Zhu T., Wang X., Heifetz P.B., Gruissem W., Lange M. (2003). Crosstalk between cytosolic and plastidial pathways of isoprenoid biosynthesis in Arabidopsis thaliana. Proc. Natl. Acad. Sci. USA.

[7] vom Dorp K., Hölzl G., Plohmann C., Eisenhut M., Abraham M., Weber A.P.M., Hanson A.D., Dörmann P. (2015). Remobilization of Phytol from Chlorophyll Degradation Is Essential for Tocopherol Synthesis and Growth of Arabidopsis. Plant Cell.

[8] Kanwischer M., Porfirova S., Bergmüller E., Dörmann P. (2005). Alterations in Tocopherol Cyclase Activity in Transgenic and Mutant Plants of Arabidopsis Affect Tocopherol Content, Tocopherol Composition, and Oxidative Stress. Plant Physiol..

[9] Surówka E., Potocka I., Dziurka M., Wróbel-Marek J., Kurczyńska E., Żur I., Maksymowicz A., Gajewska E., Miszalski Z. (2020). Tocopherols mutual balance is a key player for maintaining Arabidopsis thaliana growth under salt stress. Plant Physiol. Biochem..

[10] Wang H., Qiu C., Wang Y., Guo X. (2021). Biosynthesis and profiles of fatty acids, vitamin E and carotenoids during flax (*Linum usitatissimum* L.) seed capsule maturation. Int. J. Food Sci. Technol..

[11] Styrczewska M., Kulma A., Kostyn K., Hasiewicz-Derkacz K., Szopa J. (2013). Flax terpenoid pathway as a source of health promoting compounds. Mini-Rev. Med. Chem..

[12] Kauser S., Hussain A., Ashraf S., Fatima G., Ambreen, Javaria S., Abideen Z.U., Kabir K., Yaqub S., Akram S. (2024). Flaxseed (*Linum usitatissimum*); phytochemistry, pharmacological characteristics and functional food applications. Food Chem. Adv..

[13] Styrczewska M., Kostyn A., Kulma A., Majkowska-Skrobek G., Augustyniak D., Prescha A., Czuj T., Szopa J. (2015). Flax Fiber Hydrophobic Extract Inhibits Human Skin Cells Inflammation and Causes Remodeling of Extracellular Matrix and Wound Closure Activation. Biomed. Res. Int..

[14] Zuk M., Richter D., Matuła J., Szopa J. (2015). Linseed, the multipurpose plant. Ind. Crops Prod..

[15] Lorenc-Kukuła K., Amarowicz R., Oszmiański J., Doermann P., Starzycki M., Skała J., Zuk M., Kulma A., Szopa J. (2005). Pleiotropic effect of phenolic compounds content increases in transgenic flax plant. J. Agric. Food Chem..

[16] Ludvíková M., Griga M. (2015). Transgenic flax/linseed (*Linum usitatissimum* L.)—Expectations and reality. Czech J. Genet. Plant Breed..

[17] Lee K., Lee S.M., Park S.R., Jung J., Moon J.K., Cheong J.J., Kim M. (2007). Overexpression of Arabidopsis homogentisate phytyltransferase or tocopherol cyclase elevates vitamin E content by increasing gamma-tocopherol level in lettuce (*Lactuca sativa* L.). Mol. Cells.

[18] Szymańska R., Kruk J. (2008). Tocopherol content and isomers’ composition in selected plant species. Plant Physiol. Biochem..

[19] Liao P., Chen X., Wang M., Bach T.J., Chye M.L. (2018). Improved fruit α-tocopherol, carotenoid, squalene and phytosterol contents through manipulation of Brassica juncea 3-HYDROXY-3-METHYLGLUTARYL-COA SYNTHASE1 in transgenic tomato. Plant Biotechnol. J..

[20] Rodríguez-Concepción M., Boronat A. (2015). Breaking new ground in the regulation of the early steps of plant isoprenoid biosynthesis. Curr. Opin. Plant Biol..

[21] Fraser P.D., Enfissi E.M., Halket J.M., Truesdale M.R., Yu D., Gerrish C., Bramley P.M. (2007). Manipulation of phytoene levels in tomato fruit: Effects on isoprenoids, plastids, and intermediary metabolism. Plant Cell.

[22] Havaux M., Eymery F., Porfirova S., Rey P., Dörmann P. (2005). Vitamin E protects against photoinhibition and photooxidative stress in Arabidopsis thaliana. Plant Cell.

[23] Arnqvist L., Persson M., Jonsson L., Dutta P.C., Sitbon F. (2008). Overexpression of CYP710A1 and CYP710A4 in transgenic Arabidopsis plants increases the level of stigmasterol at the expense of sitosterol. Planta.

[24] Hey S.J., Powers S.J., Beale M.H., Hawkins N.D., Ward J.L., Halford N.G. (2006). Enhanced seed phytosterol accumulation through expression of a modified HMG-CoA reductase. Plant Biotechnol. J..

[25] Harker M., Hellyer A., Clayton J.C., Duvoix A., Lanot A., Safford R. (2003). Co-ordinate regulation of sterol biosynthesis enzyme activity during accumulation of sterols in developing rape and tobacco seed. Planta.

[26] Rippert P., Scimemi C., Dubald M., Matringe M. (2004). Engineering plant shikimate pathway for production of tocotrienol and improving herbicide resistance. Plant Physiol..

[27] Moon J.-K., Shibamoto T. (2009). Antioxidant Assays for Plant and Food Components. J. Agric. Food Chem..

[28] Maeda H., Song W., Sage T., Dellapenna D. (2014). Role of callose synthases in transfer cell wall development in tocopherol deficient Arabidopsis mutants. Front. Plant Sci..

[29] Besseau S., Hoffmann L., Geoffroy P., Lapierre C., Pollet B., Legrand M. (2007). Flavonoid accumulation in Arabidopsis repressed in lignin synthesis affects auxin transport and plant growth. Plant Cell.

[30] Boba A., Kostyn K., Kozak B., Wojtasik W., Preisner M., Prescha A., Gola E.M., Lysh D., Dudek B., Szopa J. (2020). Fusarium oxysporum infection activates the plastidial branch of the terpenoid biosynthesis pathway in flax, leading to increased ABA synthesis. Planta.

[31] Shukla V.K.S., Dutta P.C., Artz W.E. (2002). Camelina oil and its unusual cholesterol content. J. Am. Oil Chem. Soc..

[32] Li T.S.C., Beveridge T.H.J., Drover J.C.G. (2007). Phytosterol content of sea buckthorn (*Hippophae rhamnoides* L.) seed oil: Extraction and identification. Food Chem..

[33] Hrabovski N., Sinadinović-Fišer S., Nikolovski B., Sovilj M., Borota O. (2012). Phytosterols in pumpkin seed oil extracted by organic solvents and supercritical CO2. Eur. J. Lipid Sci. Technol..

[34] Mierziak J., Wojtasik W., Kostyn K., Czuj T., Szopa J., Kulma A. (2014). Crossbreeding of transgenic flax plants overproducing flavonoids and glucosyltransferase results in progeny with improved antifungal and antioxidative properties. Mol. Breed..

[35] Czemplik M., Korzun-Chłopicka U., Szatkowski M., Działo M., Szopa J., Kulma A. (2017). Optimization of Phenolic Compounds Extraction from Flax Shives and Their Effect on Human Fibroblasts. Evid. Based Complement. Altern. Med..

[36] Manganaris G.A., Vicente A.R., Crisosto C.H., Labavitch J.M. (2008). Cell wall modifications in chilling-injured plum fruit (*Prunus salicina*). Postharvest Biol. Technol..

[37] Wojtasik W., Kulma A., Dymińska L., Hanuza J., Żebrowski J., Szopa J. (2013). Fibres from flax overproducing β-1,3-glucanase show increased accumulation of pectin and phenolics and thus higher antioxidant capacity. BMC Biotechnol..

[38] Iiyama K., Wallis A.F. (1990). Determination of lignin in herbaceous plants by an improved acetyl bromide procedure. J. Sci. Food Agric..

[39] Benzie I.F., Strain J.J. (1996). The ferric reducing ability of plasma (FRAP) as a measure of “antioxidant power”: The FRAP assay. Anal. Biochem..

