# Supplementary material for: Constitutive Expression of AtVTE2 in Flax Is Associated with Changes in Isoprenoid Metabolism and Stem Cell-Wall Composition
Source: Int J Mol Sci. 2026 Sep 17;27(18):8295. doi: 10.3390/ijms27188295 (PMC13607641; doi:10.3390/ijms27188295)
Supplement: Supplementary file 1 [file ijms-27-08295-s001.zip › Fig. S1_Cellulose content.pdf]

Fig. S1

Cellulose content

Method:

The cellulose content was determined using the colourimetric method with anthrone reagent, as described by Wojtasik et al. (2013) [1]. Briefly, 20 mg of dried, ground flax straw was suspended in an acetic acid:nitric acid mixture (8:1, v/v) and incubated at 100°C for 30 min. After cooling on ice and centrifugation at 14,000 rpm for 5 min, the pellet was washed three times with 500 μL of deionized water and once with 500 μL of acetone and dried overnight at 37°C. The dried pellet was suspended in 1 mL of 81% H_2SO_4 and incubated for 1 h at room temperature with mixing. Samples were subsequently diluted, reacted with anthrone reagent for 15 min at 100°C, cooled on ice, and absorbance was measured at 620 nm. Cellulose content was calculated from a calibration curve prepared using commercial cellulose treated in parallel with the samples.

Results:

Cellulose content was evaluated in the straw of transgenic flax lines 151 and 165 and compared with the Opal control (Figure 1S). No statistically significant differences in cellulose content were observed between the transgenic lines and the control plants.

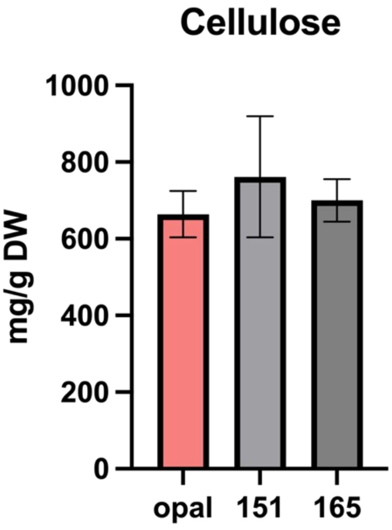

Supplementary Fig S1. Cellulose content in flax straw of transgenic lines 151 and 165 compared with the non-transgenic *Opal* control. Cellulose content is expressed per gram dry weight (DW). Data are presented as mean ± SD, n=5. Statistical significance was assessed using one-way ANOVA; *p < 0.05, **p < 0.005, and ***p < 0.001.

1. Wojtasik, W. *et al.* Fibres from flax overproducing β-1,3-glucanase show increased accumulation of pectin and phenolics and thus higher antioxidant capacity. *BMC Biotechnol.* **13**, 10 (2013).
