# Supplementary material for: Constitutive Expression of AtVTE2 in Flax Is Associated with Changes in Isoprenoid Metabolism and Stem Cell-Wall Composition
Source: Int J Mol Sci. 2026 Sep 17;27(18):8295. doi: 10.3390/ijms27188295 (PMC13607641; doi:10.3390/ijms27188295)
Supplement: Supplementary file 1 [file ijms-27-08295-s001.zip › Fig. S2_FT-IR.pdf]

Fig. S2 FT-IR analysis

Methods

Fourier-transform infrared (FTIR) spectra were acquired at room temperature over the 4000–400 cm⁻¹ spectral range using a Nicolet 6700 FT-IR spectrophotometer (Thermo Fisher Scientific, Waltham, MA, USA) at a spectral resolution of 2 cm⁻¹. The samples were analyzed as KBr pellets. Spectral acquisition and data processing were performed using OriginPro 2026 software (OriginLab Corp., Northampton, MA, USA). Before quantitative analysis, the recorded spectra were deconvolved to resolve the experimental bands into individual Lorentzian components. For spectral normalization and comparison of relative band intensities, the integral intensity of the band centered at 2925 cm⁻¹, assigned to asymmetric methyl stretching vibrations, was used as an internal reference.

Spectroscopic Analysis of Cell Wall Polymers in Studied Samples

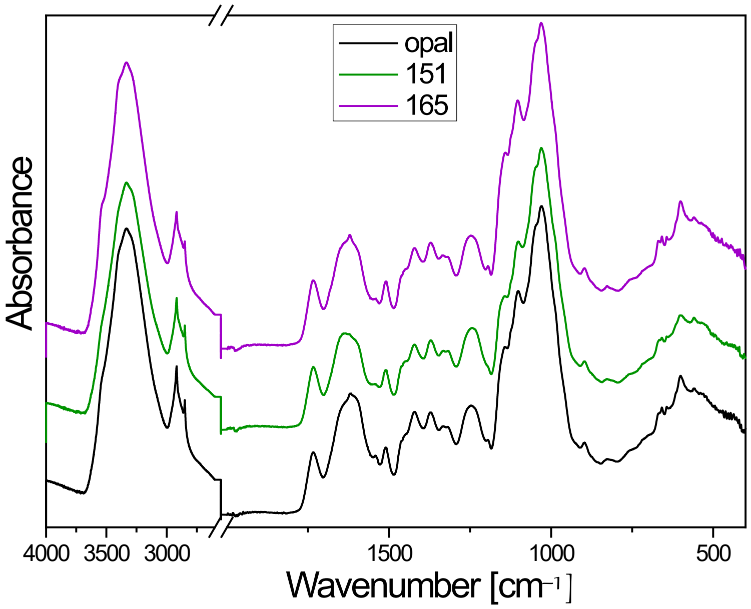

Supplementary Figure S2A. FTIR spectra of the studied samples: Opal control, lines 151 and 165.

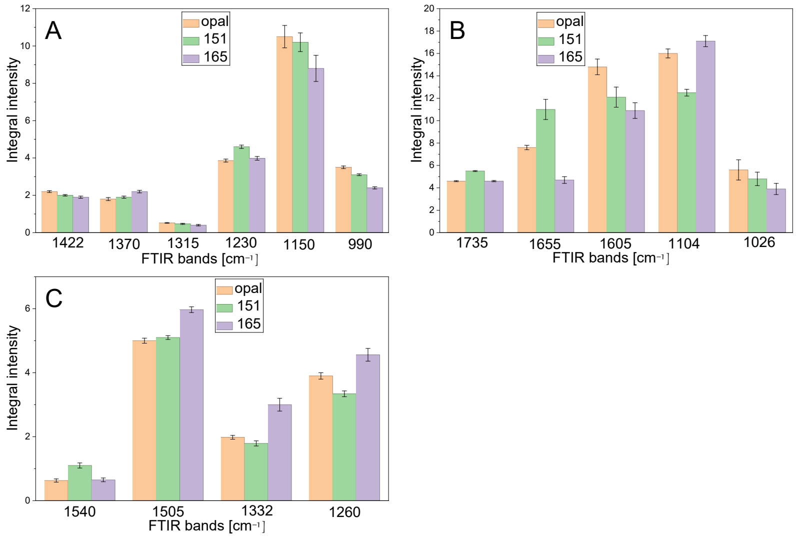

Supplementary Figure S2B. Differences in the integral intensities of FTIR absorption bands associated with characteristic cellulose (A), pectin (B), and lignin (C) bond vibrations across the studied samples.

The FTIR spectra of the three flax lines showed similar characteristic bands associated with cellulose, although differences in integral intensities were observed (Figures 1 and 2A). The bands at 1422 and 1315 cm^{-1}, assigned to CH_2 bending vibrations, showed comparable integral intensities among the lines. The band at 1370 cm^{-1}, associated with C-H bending, glucose ring stretching, and O-H deformation vibrations, increased in integral intensity from Opal to line 151 and line 165. The band at 1230 cm^{-1}, assigned to bending vibrations of hydroxyl groups involved in hydrogen bonding, showed the highest integral intensity in line 151. The bands at 1150 and 990 cm^{-1}, associated with C-O-C stretching vibrations in the cellulose glycosidic structure, exhibited the highest integral intensities in Opal. Overall, the similar cellulose spectral profiles indicate comparable cellulose characteristics and differences in selected band integral intensities, particularly at 1150 and 990 cm^{-1}, may reflect variation in cellulose structural organization and local bonding environment.

The spectra also exhibited characteristic pectin-associated bands with distinct intensity patterns. The 1735 cm^{-1} band, assigned to C=O stretching of esterified carboxyl groups, showed the highest integral intensity in line 151 (Figure 2B). The 1655 cm^{-1} band, associated with COO^- stretching vibrations, was also most intense in line 151, whereas the 1605 cm^{-1} band showed the highest integral intensity in the Opal sample. The bands at 1104 and 1026 cm^{-1}, associated with vibrations of the pectin polysaccharide backbone, showed different intensity patterns, with the highest 1104 cm^{-1} intensity in line 165 and the highest 1026 cm^{-1} intensity in the Opal sample. These differences suggest variations in the structural characteristics of pectin polysaccharides among the flax lines.

Lignin-associated bands also showed distinct differences in integral intensities. The 1540 cm^{-1} band, related to aromatic skeletal vibrations, exhibited the highest integral intensity in line 151 (Figure 2C). In contrast, line 165 showed the highest integral intensities at 1505, 1332, and 1260 cm^{-1}, which are associated with aromatic skeletal and C–O/C–C stretching vibrations in lignin. Overall, the higher integral intensities of several lignin-associated bands in lines 151 and 165 compared with Opal suggest a greater relative contribution of lignin in these lines. These differences may also reflect variations in the structural organization and chemical environment of lignin among the flax lines.
